# PRMT6-mediated transcriptional activation of ythdf2 promotes glioblastoma migration, invasion, and emt via the wnt–β-catenin pathway

**DOI:** 10.1186/s13046-024-03038-3

**Published:** 2024-04-18

**Authors:** Peng Yu, Tutu Xu, Wenmeng Ma, Xiang Fang, Yue Bao, Chengran Xu, Jinhai Huang, Yongqing Sun, Guangyu Li

**Affiliations:** 1 Department of Neurosurgery, the First Affiliated Hospital of China Medical University, No. 155, North Nanjing Street, Heping District, Shenyang, Liaoning 110001 China; 2 Department of Immunology, Basic Medicine College, China Medical University, Shenyang, Liaoning China; 3 Department of Neurosurgery, Central hospital affiliated to Shandong First Medical University, Jinan, Shandong China; 4 Department of Neurosurgery, Qingdao Municipal Hospital, Qingdao, Shandong China

**Keywords:** GBM, PRMT6, Migration, Invasion, EMT, YTHDF2, Wnt- β- catenin pathway

## Abstract

**Background:**

Protein arginine methyltransferase 6 (PRMT6) plays a crucial role in various pathophysiological processes and diseases. Glioblastoma (GBM; WHO Grade 4 glioma) is the most common and lethal primary brain tumor in adults, with a prognosis that is extremely poor, despite being less common than other systemic malignancies. Our current research finds PRMT6 upregulated in GBM, enhancing tumor malignancy. Yet, the specifics of PRMT6’s regulatory processes and potential molecular mechanisms in GBM remain largely unexplored.

**Methods:**

PRMT6’s expression and prognostic significance in GBM were assessed using glioma public databases, immunohistochemistry (IHC), and immunoblotting. Scratch and Transwell assays examined GBM cell migration and invasion. Immunoblotting evaluated the expression of epithelial-mesenchymal transition (EMT) and Wnt-β-catenin pathway-related proteins. Dual-luciferase reporter assays and ChIP-qPCR assessed the regulatory relationship between PRMT6 and YTHDF2. An in situ tumor model in nude mice evaluated in vivo conditions.

**Results:**

Bioinformatics analysis indicates high expression of PRMT6 and YTHDF2 in GBM, correlating with poor prognosis. Functional experiments show PRMT6 and YTHDF2 promote GBM migration, invasion, and EMT. Mechanistic experiments reveal PRMT6 and CDK9 co-regulate YTHDF2 expression. YTHDF2 binds and promotes the degradation of negative regulators APC and GSK3β mRNA of the Wnt-β-catenin pathway, activating it and consequently enhancing GBM malignancy.

**Conclusions:**

Our results demonstrate the PRMT6-YTHDF2-Wnt-β-Catenin axis promotes GBM migration, invasion, and EMT in vitro and in vivo, potentially serving as a therapeutic target for GBM.

**Supplementary Information:**

The online version contains supplementary material available at 10.1186/s13046-024-03038-3.

## Background

Glioma is the most common malignant tumor of the central nervous system [1]. Despite significant advancements in treatment methods, the prognosis for glioma, particularly glioblastoma multiforme, remains poor, with a median survival period of only 12–15 months [2–4]. Glioblastomas infiltrate surrounding normal brain tissue, making curative surgical resection and prevention of recurrence challenging. Various molecular markers, including isocitrate dehydrogenase 1 (IDH1), O6-methylguanine-DNA methyltransferase (MGMT), and epidermal growth factor receptor (EGFR), are currently used clinically to assess prognosis and drug sensitivity [5, 6]. Although many previous studies have described the molecular mechanisms underlying the invasive growth of gliomas [7–10], the key molecular mechanisms driving their infiltrative growth remain unclear.

The research on the regulation of gene and protein expression has always been a focal point in glioma studies. Besides the traditional regulation of transcriptional expression, the regulation of epigenetics is increasingly valued. Among them, protein arginine methylation is a widespread post-translational modification that plays a significant role in various pathophysiological processes and diseases [11]. The arginine residues of histones and other proteins can regulate DNA transcription, post-translational modifications, signal transduction, DNA repair, and mRNA splicing through methylation [12–14]. There are nine types of human protein arginine methyltransferases (PRMTs). Protein arginine methyltransferase 6 (PRMT6) belongs to the type I PRMT enzyme family. Its gene is located on chromosome 1 and encodes a protein containing the catalytic core sequence common to PRMT enzymes, capable of catalyzing the asymmetric dimethylation of arginine residues on substrates rich in glycine and arginine [15, 16]. Currently, it is found that PRMT6 mainly mediates the methylation modification of histone H3R2me2a in cells [17]. Other reported methylation sites include H2AR29me2a, H3R42me2a, and non-histone proteins such as CRAF and PTEN [14, 18–20]. PRMT6 is generally believed to play a role in transcriptional repression, and its mechanism is that H3R2me2a interferes with the formation of a complex with H3K4me3 and MLL, which have transcriptional activation effects, thus playing a role in transcriptional repression [17, 21, 22]. However, other studies have found that PRMT6 can also play a role in transcriptional activation under certain circumstances, such as acting as a co-activator of transcription factors or catalyzing the asymmetric dimethylation of arginine in the enhancer region of histone H3, etc., to activate the transcription of related genes [23–26]. In addition to methylating histones, PRMT6 can also play an important role in DNA damage repair by methylating mechanisms such as DNA polymerase β [27]. Studies have shown that PRMT6 is a new target of hypoxia, playing an important role in the aerobic glycolysis (Warburg effect) process of tumors [28]. PRMT6 can also hinder the recruitment of the auxiliary factor UHRF1 of DNA methyltransferase DNMT1 to chromatin through the methylation modification of histone H3R2me2a, thereby affecting the methylation of DNA and causing a widespread state of DNA hypomethylation in tumor cells [29]. It can be seen that PRMT6 has a wide range of important functions. However, the biological significance of PRMT6 in cancer is not yet clear. In existing reports, PRMT6 is upregulated in most tumors [30–33], but there are also reports that its expression is reduced in melanoma and hepatocellular carcinoma [20, 28, 34]. At present, there are still few studies on PRMT6 in gliomas. Existing studies have shown that PRMT6 is upregulated in gliomas and regulates the mitosis and tumorigenicity of glioblastoma stem cells by methylating RCC1, or promotes the proliferation of glioma cells by transcriptionally activating CDC20 [32, 35]. But little is known about the specific regulatory processes and potential molecular mechanisms of PRMT6 in gliomas.

m^6^A methylation modification, a vital aspect of epigenetics, is the most common in various RNA modifications and plays a critical role in the pathogenesis of cancer [36–40]. m^6^A methylation, a dynamic and reversible modification, is regulated by methyltransferases (“writers”) and demethylases (“erasers”) [41]. The mRNA modified by m^6^A can be recognized and bound by specific proteins, known as " m^6^A readers.” Among these, the YTH domain family members (YTHDF1-3, YTHDC1-2) are the main m6A readers. YTHDF2, specifically, recognizes and binds m^6^A -modified mRNA and promotes its degradation by directly recruiting the CCR4-NOT deadenylase complex or by ribonucleolytic action on m^6^A-containing RNA through the ribonuclease P/MRP complex [42–45]. Recent studies have shown that YTHDF2 plays an important role in glioma. For instance, YTHDF2 can promote the degradation of the UBXN1 gene, thereby influencing the NF-κB signaling pathway and promoting glioma progression [46]. In glioma, YTHDF2 stabilizes its protein through the EGFR/SRC/ERK signaling axis, thereby affecting tumor proliferation and other biological behaviors through LXRα-dependent cholesterol homeostasis [7]. However, in glioma stem cells, YTHDF2 promotes glioma stemness by stabilizing MYC and VEGF mRNA [47]. These studies indicate the diverse roles and complex regulatory mechanisms of YTHDF2 in glioma. However, there are no reports yet on the impact of arginine methylation on YTHDF2 expression in glioma. In this study, we found that PRMT6 promotes glioma migration, invasion, and EMT in vitro and in vivo. The related mechanism shows that in glioma, PRMT6 is recruited by the transcriptional regulator CDK9 to the YTHDF2 promoter region and then synergistically promotes the transcriptional activation of YTHDF2 with CDK9. The upregulated YTHDF2 binds to the mRNA of the negative regulatory factors APC and GSK3β in the Wnt-β-Catenin pathway that are modified by m^6^A, promoting their degradation and thereby activating the Wnt-β-Catenin pathway, ultimately promoting glioma migration, invasion, and EMT. In vitro functional experiments show that the PRMT6 small molecule inhibitor (EPZ020411) has an inhibitory effect on the migration, invasion, and EMT of glioma cells. Our research results suggest that the PRMT6-YTHDF2-Wnt-β-Catenin axis may serve as a therapeutic target for glioma.

## Materials and methods

### Clinical specimen and ethical statement

A total of 35 glioma samples (including 13 WHO grade II-III and 22 WHO grade IV) and 4 normal brain tissue (NBT) samples were collected from the Department of Neurosurgery of the First Affiliated Hospital of China Medical University. This study was approved by the Education and Ethics Committee of the First Affiliated Hospital of China Medical University, and informed consent was obtained from all participants.

### Plasmids, reagents and antibodies

The shPRMT6 plasmid and control plasmid were purchased from GeneChem (Shanghai, China). PRMT6 overexpression plasmid and empty vector were obtained from Tsingke (Beijing, China). shYTHDF2 and shCDK9 plasmids, along with their respective control plasmids, and YTHDF2 and CDK9 overexpression plasmids, were also sourced from Tsingke (Beijing, China). YTHDF2 promoter-driven luciferase reporter gene plasmid and Renilla luciferase control plasmid were acquired from OBiO Technology (Shanghai, China). EPZ020411 was purchased from Cayman (USA), CHIR-99,021 (Cat#HY-10,182 A) and Actinomycin D (Cat#HY-17,559) were from MCE (USA). The anti-PRMT6 (Cat#14,641) antibody was from CST (Boston, MA, USA). Antibodies against YTHDF2 (Cat#24744-1-AP), Cyclin D1 (Cat#60186-1-Ig), E-cadherin (Cat#20874-1-AP), and N-cadherin (Cat#22018-1-AP) were from Proteintech (Wuhan, China). Anti-β-catenin (Cat#PK02151), Vimentin (Cat#T55134) antibodies were from Abmart (Shanghai, China). Anti-p-β-catenin (Cat#sc-57,535), GSK-3β (Cat#sc-53,931) antibodies were from Santa Cruz Biotechnology (Dallas, TX, USA). Anti-APC (Cat#WL02422), c-Myc (Cat#WL01781) antibodies were from Wanleibio (Shenyang, China). Anti-Histone H3R2me2a antibodies were from Genetex (USA, Cat#GTX54134) and PTM BIO (Hangzhou, China, Cat#PTM668). Anti-CDK9 antibodies were from Abcam (UK, Cat#ab239364) and Santa Cruz Biotechnology (USA, Cat#sc-13130x).

### Bioinformatic analysis

RNA-seq data and clinical information for glioma patients from The Cancer Genome Atlas (TCGA) and normal brain tissue RNA-seq data from The Genotype-Tissue Expression (GTEx) database were both downloaded from UCSC Xena (http://xena.ucsc.edu). Additionally, mRNA expression data and clinical information for glioma patients were obtained from the Chinese Glioma Genome Atlas (CGGA, http://www.cgga.org.cn). In R Studio (R Studio Inc., Boston, MA, USA), PRMT6 and YTHDF2 expression and survival analyses were conducted. Differential gene expression analysis was performed using the Limma R package, and Gene Set Enrichment Analysis (GSEA) was conducted with the clusterProfiler R package in R Studio.

### Cell lines and cell culture conditions

Human GBM cell lines (LN229, U251MG, U87MG, U118MG), normal brain glial cell line (HEB), and HEK293T cells were obtained from the Cell Bank of the Chinese Academy of Sciences (Shanghai, China). U87MG cells were cultured in Eagle’s MEM (EMEM) supplemented with 10% fetal bovine serum at 37 °C and 5% CO2. The other cell lines were cultured in Dulbecco’s Modified Eagle Medium (DMEM) supplemented with 10% fetal bovine serum under the same conditions.

### Lentivirus packaging and infection

Lentiviral shRNA plasmids targeting PRMT6, YTHDF2, and CDK9, and plasmids expressing full-length PRMT6 and YTHDF2 were constructed. Following the manufacturer’s protocol, these plasmids were transfected into HEK-293T cells using Lipofectamine 8000 (Beyotime, China, Cat#C0533), with a plasmid ratio of pVSV-G:psPAX2:target plasmid = 1:3:4. The virus-containing supernatant was collected 36 and 48 h after transfection and used to infect target cells with 10 µg/ml polybrene (Solarbio). After 72 h of infection, cells were selected with 2 µg/mL puromycin for three days to isolate successfully infected cells. For the rescue experiments, cells were selected with 800 µg/mL G418 for 7 days to establish double-stable cell lines, in addition to the standard 2 µg/mL puromycin selection for 3 days.

### Western blot

Following the manufacturer’s instructions, cells were lysed using RIPA lysis buffer containing protease and phosphatase inhibitors (Beyotime, China, Cat# P0013B). Protein concentrations were measured using the BCA Protein Assay Kit (Beyotime, China, Cat# P0010). Equal amounts of protein were separated by SDS-PAGE (Tris-HCl) and transferred to PVDF membranes. After blocking with 5% skim milk at room temperature for 1 h, the membranes were incubated with primary antibodies overnight at 4 °C, followed by HRP-conjugated secondary antibodies at room temperature for 1 h. Enhanced Chemiluminescence (ECL) was used to detect chemiluminescent signals, which were quantified using ImageJ software (NIH, Bethesda, USA).

### Quantitative real-time PCR assay (qPCR)

Total RNA was extracted from cells using RNAiso Plus reagent (TaKaRa, Cat# 9109), as per the manufacturer’s instructions. RNA was reverse transcribed into cDNA using the abm ALL-In-One 5X RT MasterMix kit (abm, Cat# G592). The reverse-transcribed cDNA products were analyzed by qPCR using the BlasTaq™ 2X qPCR MasterMix kit (abm, Cat# G891). β-actin was used as an internal reference, and RNA expression was analyzed using the 2^−ΔΔCT^ method. Primer sequences were synthesized by BGI (Beijing, China) and are listed in Supplementary Material 8: Table S2.

### Wound healing assays

Cells were seeded in a six-well plate and incubated at 37 °C overnight until they reached 95% confluence. A sterile 10 µl pipette tip was used to gently scratch the cell surface, creating a wound. Subsequently, the cells were washed with phosphate-buffered saline to remove cell debris. To inhibit cell proliferation, the medium containing 10% fetal bovine serum was replaced with serum-free medium. Photographs were taken at 0 h, 24 h, and 48 h using an inverted microscope to record the distance of cell migration.

### Transwell assays

Transwell migration and invasion assays were conducted in a 24-well invasion chamber system (Corning, Cat#3422), with Matrigel coating (Corning, Cat#356,234) for invasion assays. To inhibit cell proliferation, we resuspended cells in serum-free culture medium (3 × 10^5^ cells/ml). Then, we added 200 µl of cell suspension to the upper chamber of the transwell, and 600 µl of culture medium containing 10% FBS to the lower chamber. After incubating at 37 °C for 18–24 h, cells in the upper chamber were fixed with 4% paraformaldehyde and stained with 0.1% crystal violet. Cells on the upper surface of the chamber were gently wiped away with a cotton swab. Photographs were taken using an inverted microscope, and cells were counted using ImageJ software.

### Co-immunoprecipitation (co-IP)

For Co-immunoprecipitation (co-IP), cells were lysed on ice for 30 min in IP lysis buffer containing protease and phosphatase inhibitors, reserving an appropriate amount of whole cell lysate as the input sample. The lysate was then pre-cleared by incubating with 15 µl of Protein G Sepharose (Cytiva, Cat#17,061,801) at 4 °C for 2 h. The pre-cleared lysate was incubated overnight with the primary antibody at 4 °C. Subsequently, 30 µl of Protein G Sepharose was added for a further 2-hour incubation at 4 °C, followed by centrifugation to collect the immunoprecipitated protein complexes. The collected protein complexes were washed three times in cold IP lysis buffer, and then subjected to Western blotting analysis alongside the input samples.

### Chromatin immunoprecipitation (ChIP) assay

For the ChIP assay, the SimpleChIP Enzymatic Chromatin IP Kit (Cell Signaling Technology, Cat# 9002 S) was used according to the manufacturer’s instructions. Briefly, cells were crosslinked with 1% formaldehyde in the culture medium, and the reaction was quenched with glycine. Cells were harvested using a scraper and lysed in ChIP lysis buffer. Chromatin was digested with micrococcal nuclease to generate DNA fragments of approximately 150–900 bp. After reserving 2% of the sample as input, the remaining chromatin solution was incubated with the specific primary antibody or IgG negative control overnight at 4 °C. Protein G Agarose Beads were added to the chromatin solution and incubated for 2 h at 4 °C. The chromatin was then eluted from the antibody/Protein G Agarose Beads and de-crosslinked. Finally, DNA was purified and quantified by qPCR. Primers used for ChIP assays are listed in Supplementary Material 8: Table S2.

### Luciferase reporter assay

For the dual-luciferase reporter assay, a YTHDF2 promoter-driven luciferase reporter gene plasmid was constructed and transfected into HEK293T or glioma cell lines. Twenty-four hours post-transfection, luciferase activity was measured using the Dual-Luciferase Reporter Assay System (Promega, Cat# E1910). Renilla luciferase activity was used to normalize the firefly luciferase activity.

### RNA immunoprecipitation (RIP)assays

For the RIP experiment, we used the EZ-Magna RIP RNA-Binding Protein Immunoprecipitation Kit (Millipore, Cat#17–701) following the manufacturer’s instructions. Cells were collected using a scraper and lysed with RIP lysis buffer. Protein A/G magnetic beads were washed with RIP wash buffer and incubated with 5 µg of specific antibody or negative control IgG at room temperature for 30 min. The RIP lysate was then added to the antibody-bead complex (reserving 10% of the lysate as input, stored at -80 °C) and incubated overnight at 4 °C. After collection with a magnetic stand and discarding the supernatant, proteinase K buffer was added to elute from the beads. The supernatant was transferred to a new tube, and RNA was extracted with RNAiso plus. The RNA was reverse transcribed to cDNA and quantified by qPCR. Primers used for RIP are listed in Supplementary Material 8: Table S2.

### MeRIP-qPCR

Total RNA was extracted from cells overexpressing PRMT6 or YTHDF2. The extracted RNA was fragmented using RNA Fragmentation Reagents (Invitrogen, Cat#AM8740) to approximately 300nt in size, and fragmented RNA was recovered using an RNA purification column (Zymo Research, Cat#R1017). BSA was mixed with Protein A/G magnetic beads and rotated at 4 °C for 2 h to block the beads. 50 µg of fragmented RNA was then mixed with 5 µg of m^6^A antibody or IgG control and incubated with the blocked beads overnight at 4 °C. After collecting the beads with a magnetic stand and discarding the supernatant, the bound RNA was eluted and digested with proteinase K. The eluted RNA was extracted using an RNA purification column, reverse transcribed, and quantitatively analyzed by qPCR. Primers for MeRIP experiments are listed in Supplementary Material 8: Table S2.

### Immunohistochemistry (IHC)

Tissues were fixed with 4% paraformaldehyde, paraffin-embedded, and sectioned. The paraffin sections were routinely deparaffinized, rehydrated, and underwent antigen retrieval. Staining was performed using the KeyGEN One-Step IHC Assay Kit (KeyGEN, Cat#KGOS60), following the manufacturer’s instructions. The immunohistochemical (IHC) staining scores were evaluated using the Immunoreactive Score (IRS) system, as described in previous literature [7].

### mRNA stability assay

The corresponding cells were seeded in six-well plates and treated with 5 µM Actinomycin D (MCE, Cat#HY-17,559) for 0, 1, 3, and 6 h. Total RNA from each sample was extracted using RNAiso Plus for qPCR analysis. The half-life (t_1/2_) of mRNA was calculated using the formula: t_1/2_ = ln 2 / k_decay_, as previously reported [48].

### Tumor xenograft in nude mouse

Four-week-old female athymic BALB/c nude mice were purchased from SPF Biotechnology Co., Ltd. (Beijing, China) and acclimatized for one week. The mice were randomly divided into three groups, with five in each group. LN229 cells (5 × 10^5^) suspended in 3 µl phosphate-buffered saline were injected into the mice’s brain using a microsyringe. To analyze tumor growth and invasion, brain tissues were collected 35 days post-injection, fixed in 4% paraformaldehyde, and paraffin-embedded for Hematoxylin and Eosin (H&E) and immunohistochemistry (IHC) staining. Tumor volume and relative invasive fingers were evaluated based on previous literature [7]. Tumor volume was calculated using the formula V = L × W^2^/2, where L is the tumor length and W is the width. The relative invasive fingers of each tumor was estimated under a microscope by counting protruding and diffused tumor tissues. In the in vivo experiments with PRMT6 inhibitor, according to the dosage described in previous literature [32], mice in the EPZ020411 treatment group were subcutaneously administered with EPZ020411 at a dose of 10 mg/kg daily for 3 weeks, while the control group received an equal volume of saline solution.

### Statistical analysis

Statistical analysis was conducted using GraphPad Prism 8.3 and R studio software. All experiments were repeated at least three times, and results are presented as mean ± standard deviation (SD). The Kruskal-Wallis test, Wilcoxon rank-sum test, and t-test were used to evaluate statistical significance between groups. Differences in survival rates were analyzed using Kaplan-Meier analysis and log-rank tests. Pearson or Spearman methods were applied to assess correlations in gene expression. A P-value < 0.05 was considered statistically significant.

## Results

### PRMT6 is highly expressed in gliomas and is associated with poor prognosis

**Fig. 1:**
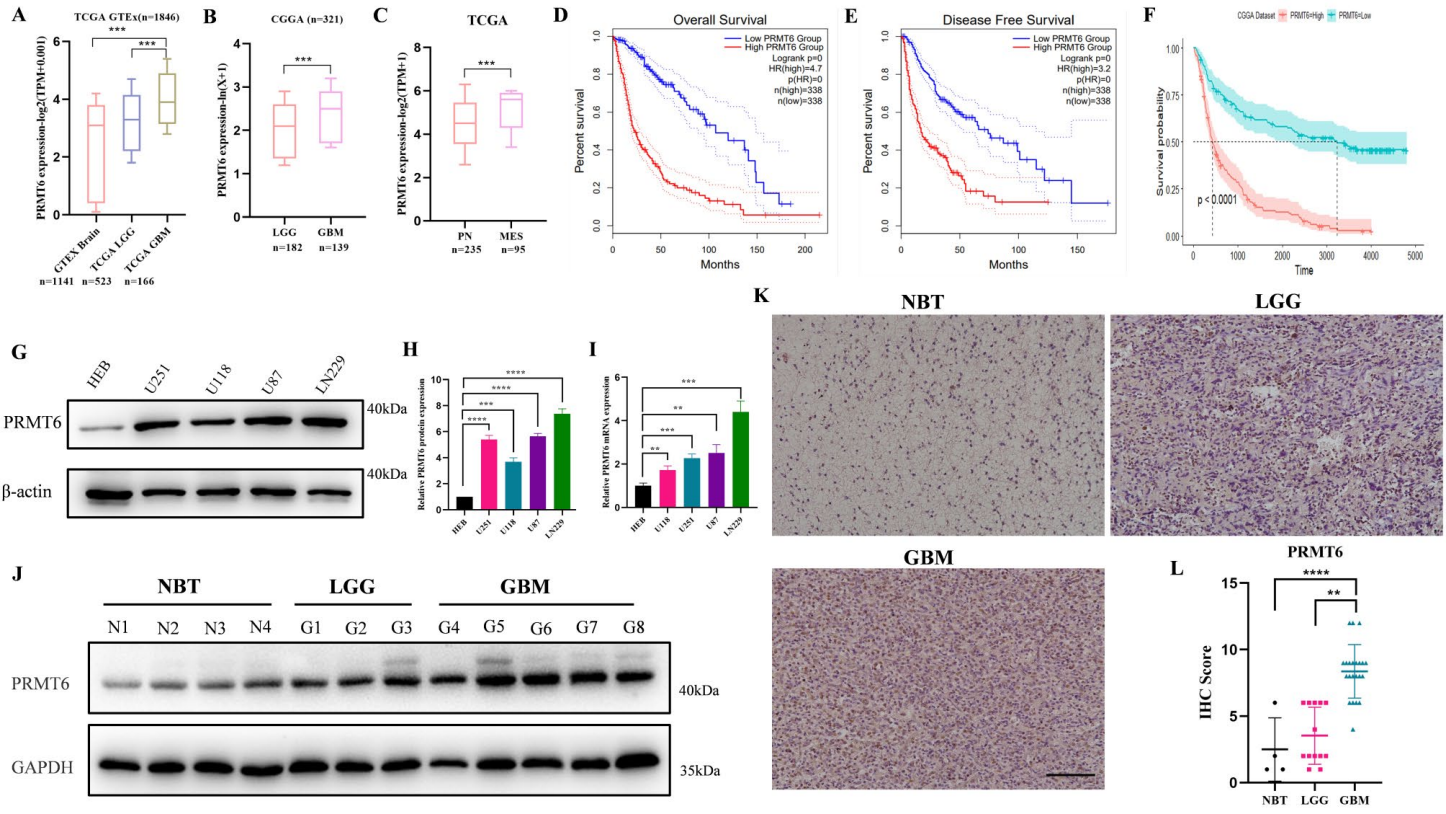
Elevated Expression of PRMT6 in Glioma and Its Association with Poor Prognosis. **A-C**: Analysis of PRMT6 mRNA expression in various grades and subtypes of glioma using data from TCGA and CGGA databases. **D-E**: Kaplan-Meier survival analysis showing overall and progression-free survival of glioma patients with high and low PRMT6 expression in the TCGA dataset. **F**: Kaplan-Meier survival analysis of overall survival in glioma patients with high and low PRMT6 expression in the CGGA dataset. **G-I**: Analysis of PRMT6 protein and mRNA expression in normal human brain glial cell line (HEB) and glioma cell lines (LN229, U118, U87, U251). **J**: Clinical sample collection and analysis of PRMT6 expression in different grades of glioma patients and normal brain tissue (NBT). **K-L**: Representative immunohistochemistry images of PRMT6 in different grades of glioma patients and NBT. Kruskal-Wallis test and Dunn’s multiple comparisons test were used. Scale bar = 100 μm

To understand the role of PRMT6 in glioma, we first explored its expression spectrum and prognostic significance in public databases such as TCGA, CGGA, and GTEXBrain. In the TCGA and CGGA databases, we found that PRMT6 expression increases with tumor grade (Fig. 1A-B). Compared to tumor tissues, PRMT6 expression is significantly lower in normal brain tissues recorded in the GTEXBrain database (Fig. 1A). Previous research classified glioblastoma into three subtypes: Mesenchymal (MES), Classical (CL), and Proneural (PN) [49]. Compared to the PN subtype, MES subtype glioblastomas, which are more aggressive and have poorer prognosis, exhibit significantly higher PRMT6 expression levels (Fig. 1C). We then explored the prognostic significance of PRMT6 in glioblastoma patients. Kaplan-Meier survival analysis revealed that patients with high PRMT6 expression have shorter overall and progression-free survival compared to those with low PRMT6 expression (Fig. 1D-E). Similar results were observed in the CGGA dataset (Fig. 1F). Western blot and Quantitative Real-Time PCR (qPCR) analyses show that, at both mRNA and protein levels, PRMT6 expression is higher in glioblastoma cell lines compared to normal human brain glial cells (HEB) (Fig. 1G-I). Furthermore, Western blot results of freshly collected glioblastoma patient protein samples show that PRMT6 protein level expression is significantly higher in glioma, especially in GBM, compared to Normal Brain Tissue (NBT) (Fig. 1J). Immunohistochemistry (IHC) results also demonstrate that PRMT6 expression increases with tumor grade in glioma (Fig. 1K-L). In summary, high expression of PRMT6 is associated with poor prognosis and may promote the progression of glioblastoma.

### PRMT6 promotes migration, invasion, and EMT in glioblastoma

**Fig. 2:**
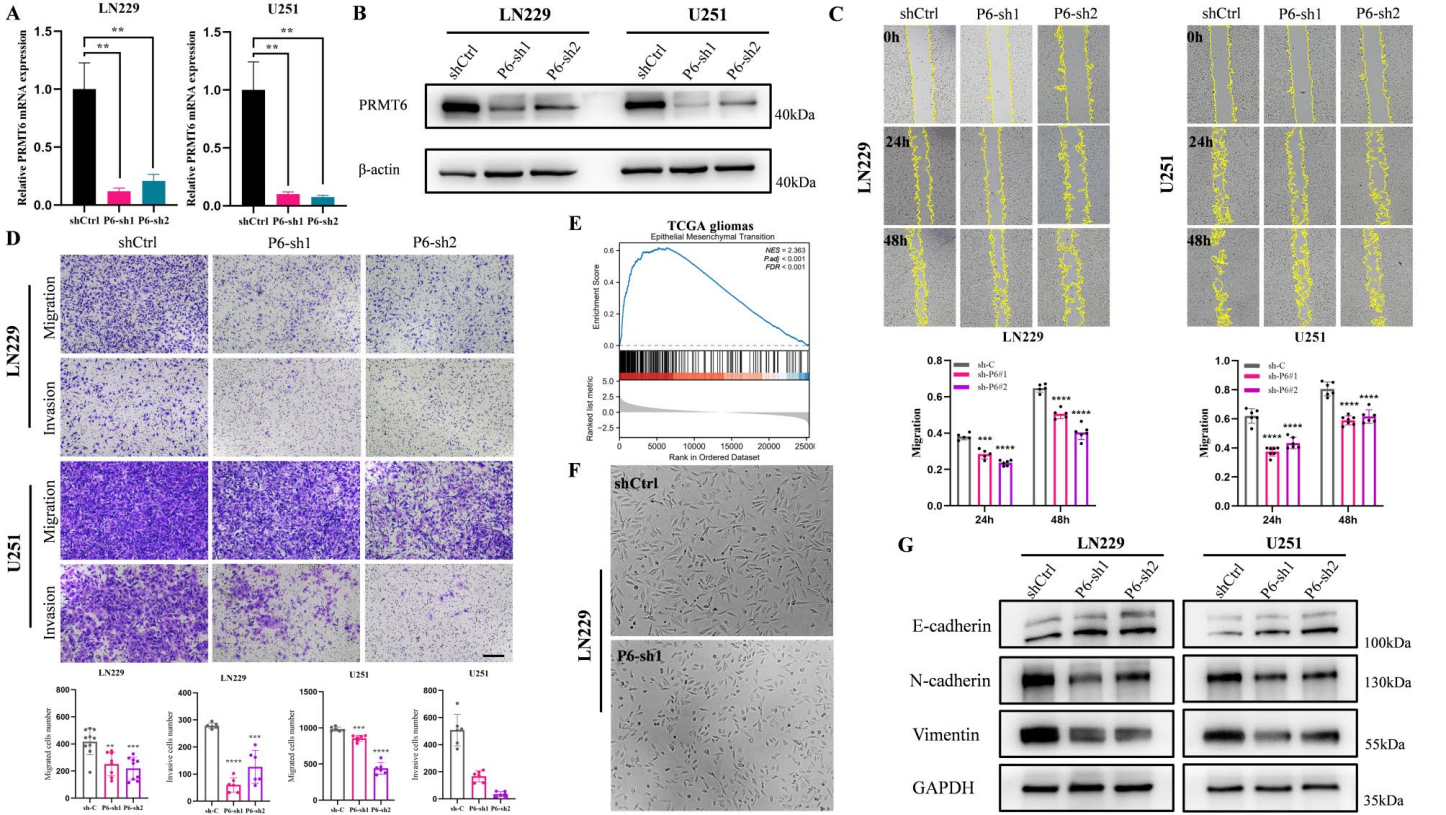
Knockdown of PRMT6 Inhibits Migration, Invasion, and EMT in Glioma. **A-B**: RNA and protein expression of PRMT6 were analyzed in LN229 and U251 cell lines infected with PRMT6 shRNA or scramble shRNA lentivirus. **C**: Wound healing assay to evaluate the migration of LN229 and U251 cells with or without PRMT6 knockdown. **D**: Transwell assay assessing migration and invasion of LN229 and U251 cells with or without PRMT6 knockdown. Scale bar = 200 μm. **E**: Gene Set Enrichment Analysis (GSEA) based on PRMT6 expression in TCGA glioma dataset, suggesting PRMT6 promotes Epithelial-Mesenchymal Transition (EMT). **F**: In LN229 cells, PRMT6 knockdown leads to morphological changes resembling epithelial cell transformation, with rounder shape and fewer pseudopodia. **G**: Expression of EMT-related proteins E-cadherin, N-cadherin, and Vimentin in LN229 and U251 cells with or without PRMT6 knockdown

To explore the effect of PRMT6 on the malignancy of glioblastoma, we utilized lentiviruses carrying PRMT6 shRNA and overexpression plasmids to create cell lines with either decreased or increased PRMT6 expression. We developed PRMT6 knockdown models in LN229 and U251 cell lines, which have relatively high baseline PRMT6 expression, and overexpression models in U118 and U87 cell lines, where PRMT6 is normally less expressed (Fig. 1G-H). Western blot and qPCR results showed that PRMT6 mRNA and protein expression were significantly reduced in LN229 and U251, while increased in U87 and U118 (Fig. 2A-B, Supplementary Material 1: Fig. S1A-B). Subsequent wound healing assays indicated that silencing PRMT6 markedly inhibited cell migration in LN229 and U251, and overexpression of PRMT6 enhanced migration in U118 and U87 (Fig. 2C, Supplementary Material 1: Fig. S1C). In further transwell migration and invasion experiments, the number of migrating and invading cells in the PRMT6 knockdown group was lower than the control in LN229 and U251 (Fig. 2D), whereas in U118 and U87, PRMT6 overexpression led to a higher number of migrating and invading cells compared to the vector group (Supplementary Material 1: Fig. S1D). Epithelial-Mesenchymal Transition (EMT) is a biological process that enables polarized epithelial cells to acquire mesenchymal characteristics, thereby facilitating tumor migration and invasion [50, 51]. Thus, we investigated whether PRMT6 expression affects EMT. Using gene set enrichment analysis (GSEA) in TCGA and CGGA databases, we found that genes highly expressed in the PRMT6 high-expression group were enriched in EMT, suggesting that high PRMT6 expression may promote this biological process (Fig. 2E, Supplementary Material 1: Fig.S1E). Indeed, knocking down PRMT6 led to noticeable changes in cell morphology, transitioning from a spindle-like to a more round and shield-like shape, with fewer pseudopodia (Fig. 2F). Western blot results indicated that knocking down PRMT6 significantly reduced the expression of mesenchymal markers Vimentin and N-cadherin but increased epithelial marker E-cadherin expression in LN299 and U251 (Fig. 2G). Conversely, overexpressing PRMT6 increased the expression of Vimentin and N-cadherin, and decreased E-cadherin expression in U87 and U118 (Supplementary Material 1: Fig. S1F). The above experimental results suggest that PRMT6 may promote the migration, invasion, and EMT of glioblastoma cells.

### PRMT6 is associated with the m^6^A reader protein YTHDF2 and promotes its expression

**Fig. 3:**
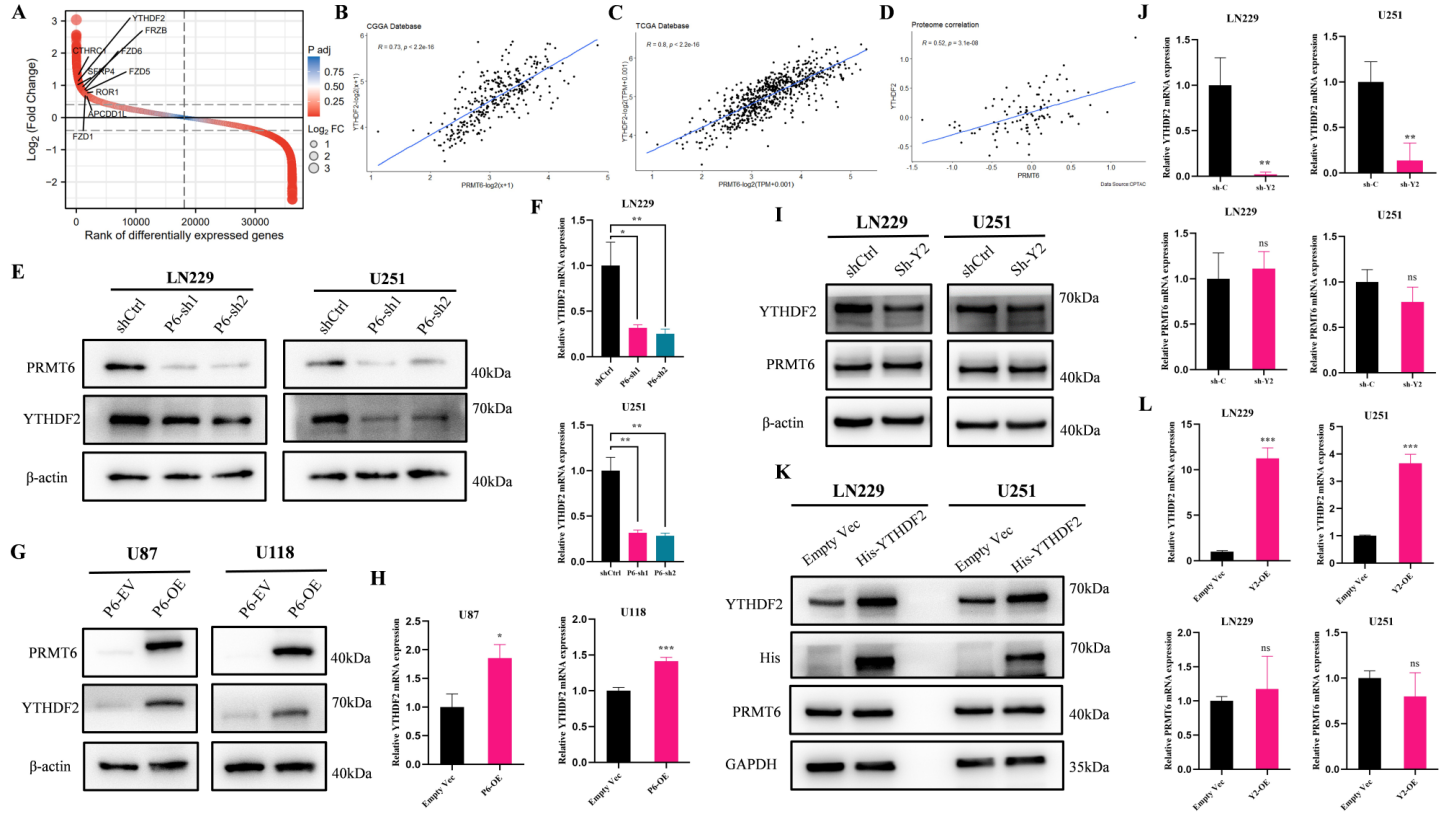
PRMT6 Is Associated with YTHDF2 and Promotes Its Expression. **A**: Differential analysis based on PRMT6 mRNA expression levels in glioma samples from the CGGA database, categorized into “high PRMT6 expression” and “low PRMT6 expression” groups. **B**: Correlation analysis of PRMT6 and YTHDF2 using mRNA sequencing data from glioma in the CGGA database. **C**: Correlation analysis of PRMT6 and YTHDF2 in glioma using mRNA sequencing data from the TCGA database. **D**: Correlation analysis of PRMT6 and YTHDF2 in glioma using protein data from the CPTAC database. **E-F**: Examination of YTHDF2 protein and mRNA expression in LN229 and U251 cells with or without PRMT6 knockdown. G-H: Analysis of YTHDF2 protein and mRNA expression in U87 and U118 cells with or without PRMT6 overexpression. I-J: Evaluation of PRMT6 protein and mRNA expression in LN229 and U251 cells with or without YTHDF2 knockdown. K-L: Assessment of PRMT6 protein and mRNA expression in LN229 and U251 cells with or without YTHDF2 overexpression

To explore how PRMT6 enhances glioblastoma’s malignant behaviors such as invasion, migration, and EMT, we sought to identify potential downstream targets of PRMT6 in glioblastoma cells. Samples in the CGGA glioma database were categorized into “high PRMT6 expression” and “low PRMT6 expression” groups based on PRMT6 mRNA levels. Following differential analysis with |log2(FC)|>1 and adj P Value < 0.05 as cutoffs, 3131 differentially expressed genes were identified (Fig. 3A). Correlation analysis between all differentially expressed genes and PRMT6 revealed that the m^6^A reader protein YTHDF2 had the highest correlation with PRMT6 (Supplementary Material 7: Table S1), showing a strong positive correlation (Fig. 3B), while other members of the YTH domain family showed lower correlation ( Supplementary Material 2: Fig. S2A). In the TCGA database, PRMT6 also showed a strong positive correlation with YTHDF2 (Fig. 3C), but lower correlations with other YTH domain family members (Supplementary Material 2: Fig. S2B). At the protein level in the CPTAC proteomics database, PRMT6 and YTHDF2 also demonstrated a strong positive correlation in GBM patients (Fig. 3D), with lower correlations with other YTH domain family molecules (Supplementary Material 2: Fig. S2C), suggesting a potential influence of PRMT6 on YTHDF2 expression. After knocking down or overexpressing PRMT6, YTHDF2 expression also decreased or increased, respectively, both at mRNA and protein levels (Fig. 3E-H). However, manipulating YTHDF2 expression did not significantly affect PRMT6 levels (Fig. 3I-L), indicating that PRMT6 might regulate YTHDF2 at the transcriptional level.

### PRMT6 interacts with CDK9 to regulate YTHDF2 expression

**Fig. 4:**
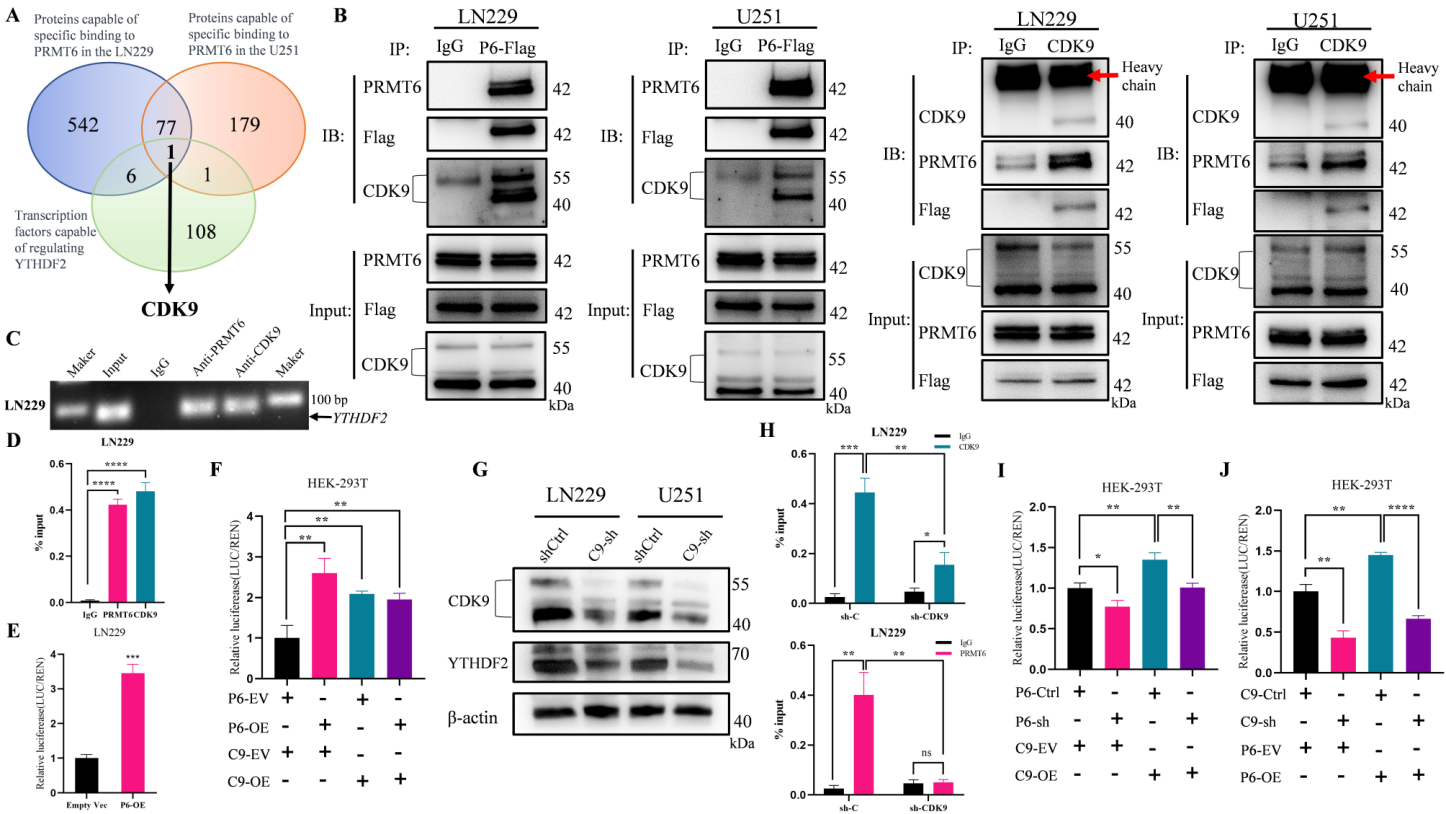
PRMT6 Interacts with CDK9 to Co-Regulate YTHDF2 Expression. **A**: Proteins specifically binding to PRMT6 in LN229 and U251 cell lines identified through mass spectrometry, intersected with transcription factors regulating YTHDF2 from the Cistrome database. **B**: Co-IP assay assessing the interaction between PRMT6 and CDK9 in LN229 and U251 cells. **C-D**: ChIP-PCR and ChIP-qPCR confirming the binding of PRMT6 and CDK9 to the YTHDF2 promoter region in LN229 cells. **E**: Dual-luciferase reporter assay in LN229 cells transfected with a YTHDF2 promoter-driven luciferase reporter plasmid, evaluating luciferase activity post overexpression or non-overexpression of PRMT6. **F**: Dual-luciferase reporter assay in HEK-293T cells, assessing luciferase activity after single or combined overexpression of PRMT6 and CDK9. G: Evaluation of CDK9 and YTHDF2 protein expression in LN229 and U251 cells with or without CDK9 knockdown. **H**: ChIP-qPCR analysis of CDK9 and PRMT6 binding to the YTHDF2 promoter region in LN229 cells with or without CDK9 knockdown. I: Dual-luciferase reporter assay in HEK-293T cells, measuring luciferase activity with PRMT6 knockdown, CDK9 overexpression, or CDK9 overexpression on a background of PRMT6 knockdown. **J**: Dual-luciferase reporter assay in HEK-293T cells, testing luciferase activity with CDK9 knockdown, PRMT6 overexpression, or PRMT6 overexpression on a background of CDK9 knockdown

Our experimental findings suggest that PRMT6 may promote YTHDF2 expression at the transcriptional level. PRMTs, lacking the capability to bind directly to DNA, are recruited to target genes via transcription factors and participate in multi-component transcription complexes, thus activating target gene expression [24, 25]. To identify the transcription factor that recruits PRMT6 to its target gene, we employed mass spectrometry to determine PRMT6’s interacting proteins. In the LN229 cell line, we identified 626 proteins specifically binding to PRMT6, and in the U251 cell line, 258 such proteins. Combining these datasets left 78 proteins that specifically interact with PRMT6 (Fig. 4A). Using the Cistrome DB (http://cistrome.org/db/#/), which contains extensive data on human and mouse transcription factors, histone modifications, and chromatin accessibility, we identified 116 transcription factors potentially regulating YTHDF2. We then intersected these with the proteins specifically binding to PRMT6, ultimately identifying CDK9 as the potential transcriptional regulator (Fig. 4A). Indeed, CDK9 ChIP-seq data from the Cistrome database revealed a significant enrichment peak of CDK9 at the YTHDF2 promoter region (Supplementary Material 3: Fig. S3A), leading us to hypothesize that CDK9 might recruit PRMT6 to the YTHDF2 promoter, thus co-regulating YTHDF2 expression. Subsequent Co-IP experiments in glioblastoma cell lines confirmed the interaction between PRMT6 and CDK9 (Fig. 4B,Supplementary Material 3: Fig. S3B). Further ChIP-qPCR experiments indicated that both PRMT6 and CDK9 could bind to the YTHDF2 promoter region (Fig. 4C-D,Supplementary Material 3: Fig. S3C-D). In dual-luciferase reporter gene assays, we found an increase in luciferase activity following the overexpression of PRMT6 in LN229 glioblastoma cell lines (Fig. 4E). These findings suggest that PRMT6 can bind to the YTHDF2 promoter region and enhance its transcriptional activation. To explore further the synergistic effect of PRMT6 and CDK9 in promoting YTHDF2 expression, we conducted dual-luciferase reporter gene assays in HEK-293T cells with simultaneous overexpression of both PRMT6 and CDK9. We found that overexpressing either PRMT6 or CDK9 alone indeed increased luciferase activity, but simultaneous overexpression of both did not yield an additive effect (Fig. 4F). Next, we constructed stable cell lines with silenced CDK9 using shRNA lentivirus and found that knocking down CDK9 led to a decrease in YTHDF2 expression at both mRNA and protein levels (Fig. 4G, Supplementary Material 3: Fig. S3E). Further ChIP-qPCR experiments revealed that CDK9 knockdown reduced its binding to the YTHDF2 promoter region, and likewise, PRMT6 binding decreased (Fig. 4H, Supplementary Material 3: Fig. S3F). These results support the hypothesis that CDK9 can recruit PRMT6 to the YTHDF2 promoter region. Further dual-luciferase reporter gene assays showed that knocking down PRMT6 alone or overexpressing CDK9 indeed decreases or increases luciferase activity, respectively (Fig. 4I). However, compared to CDK9 overexpression alone, when CDK9 overexpression was combined with PRMT6 knockdown, luciferase activity decreased (Fig. 4I). Similar experimental results were observed when PRMT6 was overexpressed on the basis of CDK9 knockdown (Fig. 4J). These results collectively suggest that PRMT6 is recruited by CDK9 to the YTHDF2 promoter region and that PRMT6 collaborates with CDK9 to promote the transcriptional activation of YTHDF2.

### YTHDF2 in glioblastoma elevated expression promoting migration, invasion, and EMT, and correlated with poor prognosis

**Fig. 5:**
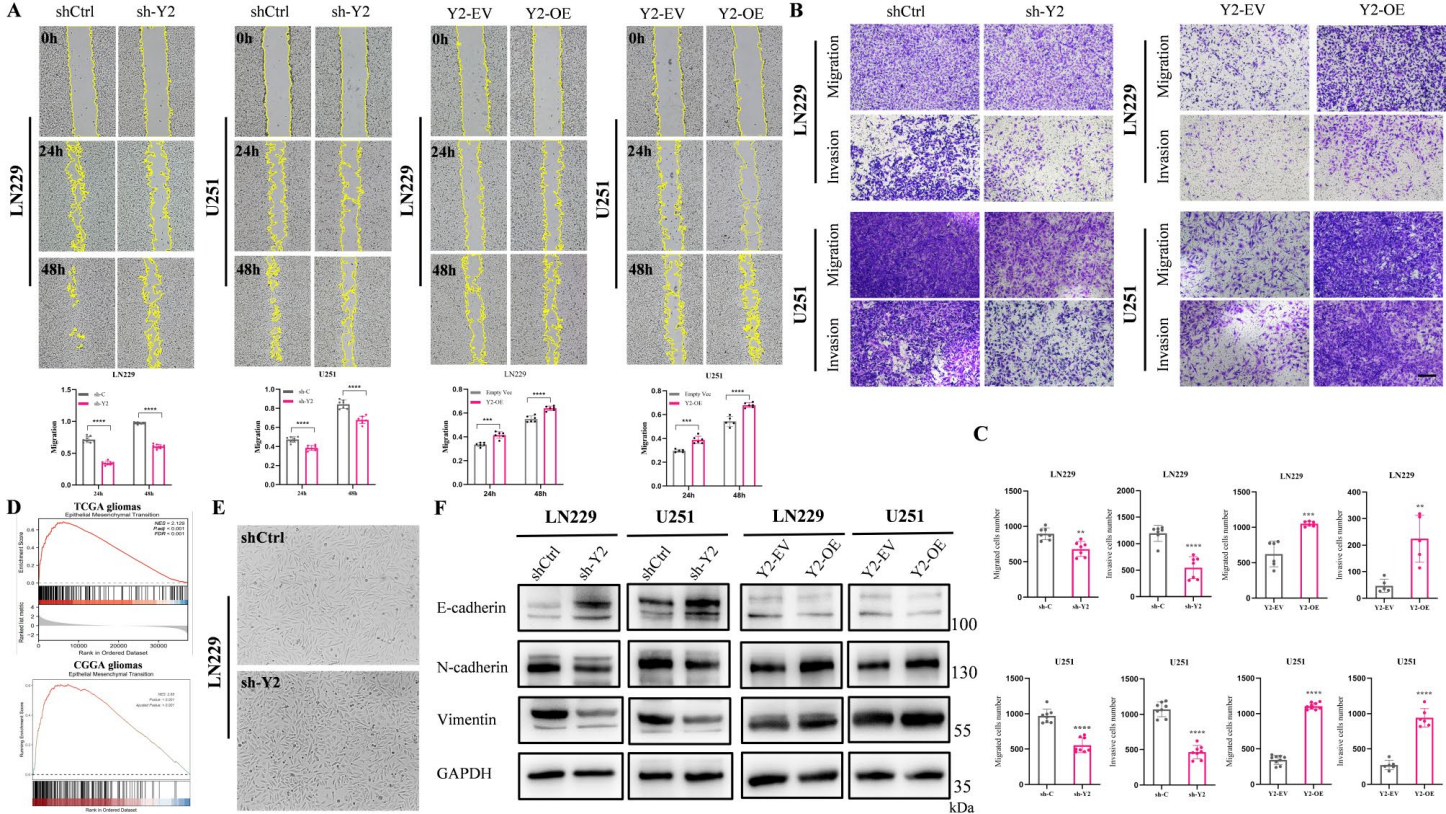
YTHDF2 Promotes Migration, Invasion, and EMT in Glioma. **A**: Scratch assay evaluating the migration rate of LN229 and U251 cells with knocked-down or overexpressed YTHDF2. **B-C**: Transwell assay assessing the migration and invasion of LN229 and U251 cells with YTHDF2 knockdown or overexpression. Scale bar = 200 μm. **D**: GSEA analysis using glioma datasets from TCGA and CGGA databases, grouped by YTHDF2 expression, indicating YTHDF2’s promotion of Epithelial-Mesenchymal Transition (EMT). **E**: Morphological changes in LN229 cells resembling epithelial characteristics post YTHDF2 knockdown, including rounder shape and reduced pseudopodia. **F**: Evaluation of EMT-related proteins E-cadherin, N-cadherin, and Vimentin in LN229 and U251 cells with YTHDF2 knockdown or overexpression

To clarify YTHDF2’s role in glioblastoma progression, we revisited its expression and prognostic data in public databases like TCGA and CGGA. We noted that YTHDF2 expression increases with tumor grade (Supplementary Material 4: Fig. S4A-B). In glioblastoma subtypes, MES subtype patients showed significantly higher YTHDF2 levels compared to PN subtype (Supplementary Material 4: Fig. S4C). Kaplan-Meier survival analysis in TCGA and CGGA databases indicated poor prognosis for patients with high YTHDF2 expression (Supplementary Material 4: Fig. S4D-F). At both mRNA and protein levels, YTHDF2 expression was higher in glioblastoma cell lines compared to normal human brain glial cells (HEB) (Supplementary Material 4: Fig. S4G-H). Immunohistochemistry (IHC) of clinical samples revealed that YTHDF2 expression increases with tumor grade and correlates positively with PRMT6 expression (Supplementary Material 4: Fig. S4I-K). Previous research has shown PRMT6 enhances glioblastoma cell migration, invasion, and EMT. Since YTHDF2 is a downstream target of PRMT6, we investigated whether YTHDF2 also promotes these malignant phenotypes. We constructed stable cell lines with knocked-down or overexpressed YTHDF2 (Fig. 3I-L). Wound healing assays showed that silencing YTHDF2 significantly inhibited, while overexpressing YTHDF2 enhanced, glioblastoma cell migration (Fig. 5A). Transwell migration and invasion assays confirmed this: cells with YTHDF2 knockdown showed fewer migrating and invading cells compared to controls, whereas cells with YTHDF2 overexpression showed more (Fig. 5B-C). GSEA pathway analysis using TCGA and CGGA databases suggested that genes highly expressed in the YTHDF2 high-expression group are enriched in the EMT process, indicating that YTHDF2 might also promote EMT in glioblastoma (Fig. 5D). In the LN229 cell line, YTHDF2 knockdown resulted in a morphological change similar to that seen with PRMT6 knockdown, with cells showing fewer pseudopodia and more epithelial-like characteristics (Fig. 5E). Immunoblotting revealed that knocking down YTHDF2 significantly decreased the expression of mesenchymal markers Vimentin and N-cadherin while increasing the epithelial marker E-cadherin (Fig. 5F). Overexpressing YTHDF2 produced the opposite effect (Fig. 5F). In summary, YTHDF2 is highly expressed in glioblastoma, correlates with poor prognosis, and promotes malignant progression of the disease.

### PRMT6 promotes glioblastoma migration, invasion, and emt dependent on YTHDF2

**Fig. 6:**
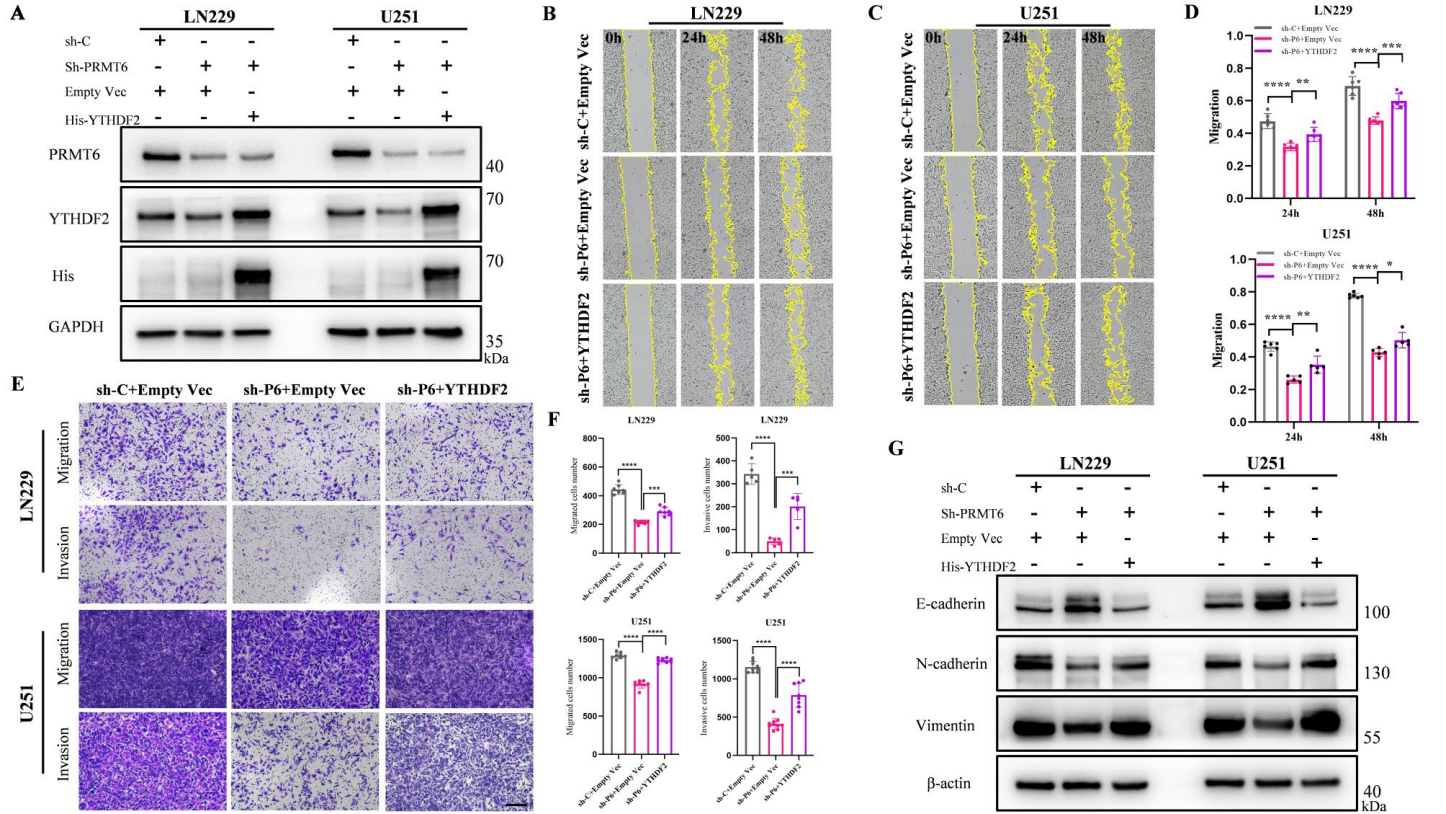
PRMT6 Facilitates Migration, Invasion, and EMT in Glioma via YTHDF2. **A**: Protein expression analysis of PRMT6 and YTHDF2 in LN229 and U251 cell lines, where YTHDF2 is reintroduced in a PRMT6 knockdown context. **B-D**: Scratch assays to assess migration rates in LN229 and U251 cells upon YTHDF2 rescue in the PRMT6 knockdown background. **E-F**: Transwell assays to evaluate cell migration and invasion following YTHDF2 re-expression in PRMT6 knockdown LN229 and U251 cells. Scale bar = 200 μm. **G**: Assessment of EMT marker proteins E-cadherin, N-cadherin, and Vimentin in LN229 and U251 cells post-rescue of YTHDF2 on a PRMT6 knockdown framework

To confirm the role of YTHDF2 in PRMT6-induced malignant phenotypes in glioblastoma, we used YTHDF2-overexpressing lentivirus to infect PRMT6-knockdown stable cell lines, creating a double-stable cell model for rescue experiments (Fig. 6A). We found that overexpression of YTHDF2 partially restored the migration and invasion capabilities inhibited by PRMT6 knockdown (Fig. 6B-F). Additionally, PRMT6 knockdown was found to inhibit EMT, but re-expression of YTHDF2 on a PRMT6-knockdown background largely restored the EMT process (Fig. 6G). These results confirm that PRMT6 regulates the malignant progression of glioblastoma through YTHDF2.

### PRMT6 regulates Wnt-β-catenin pathway activation via YTHDF2

**Fig. 7:**
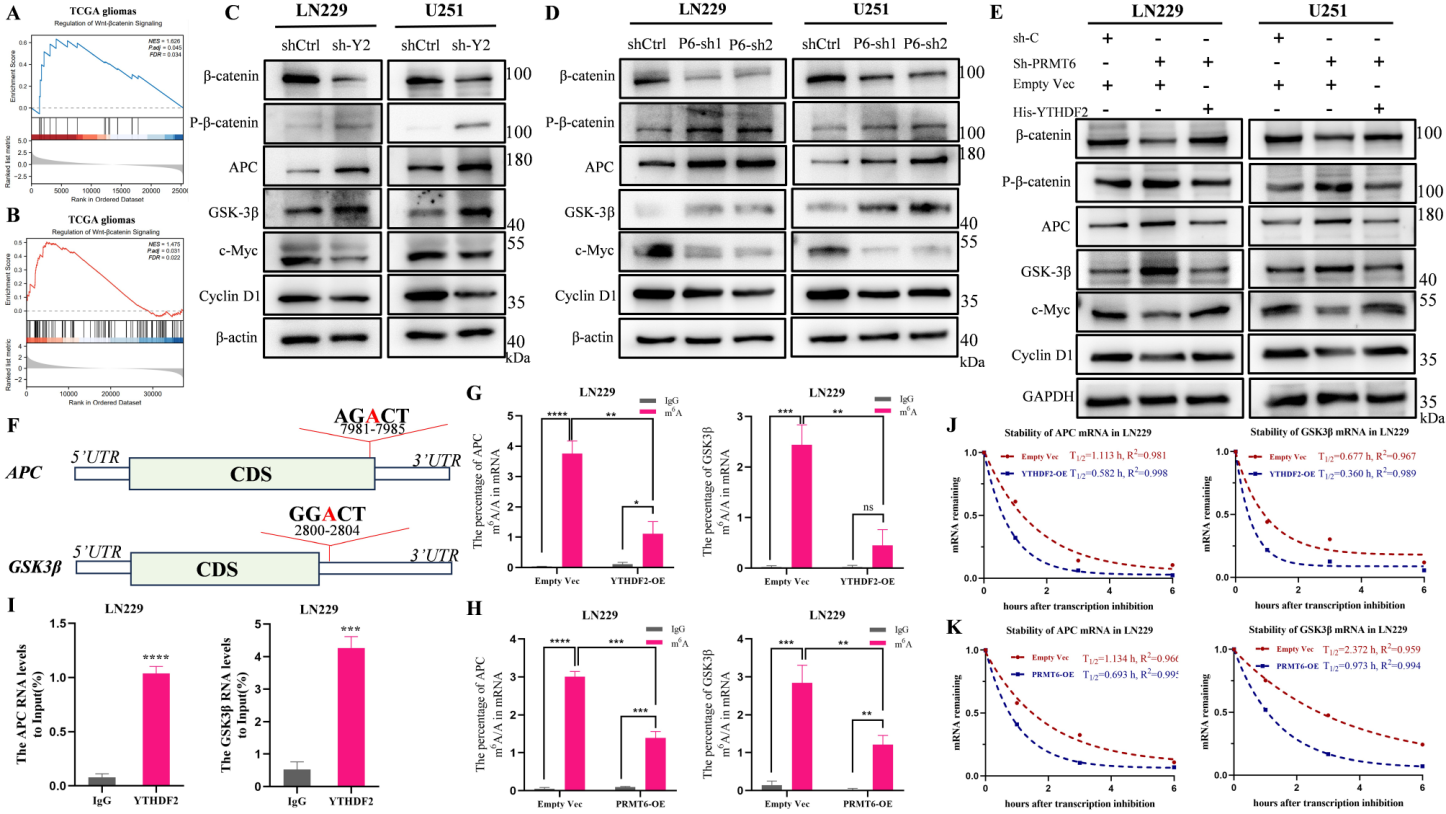
PRMT6 Modulates Wnt-β-Catenin Pathway Activation via YTHDF2. **A**: GSEA in glioma samples from TCGA database, categorizing based on PRMT6 expression, indicates PRMT6 activation of Wnt-β-catenin pathway. **B**: Similar GSEA, based on YTHDF2 expression in TCGA database, suggests YTHDF2’s role in activating Wnt-β-catenin pathway. **C-D**: Analysis of Wnt-β-catenin pathway proteins in YTHDF2 or PRMT6 knockdown LN229 and U251 cells. **F**: YTHDF2 reintroduction in PRMT6 knockdown cells and its effect on Wnt-β-catenin pathway proteins. **F**: Illustration of m^6^A modification sites near the 3’ end of APC and GSK3β mRNA, potential YTHDF2 targets. **G-H**: MeRIP-qPCR analysis in LN229 cells to assess m^6^A modification levels near the 3’ end of APC and GSK3β mRNA, potentially recognized by YTHDF2, in the context of overexpression or normal expression of YTHDF2 and PRMT6, respectively. **I**: RIP-qPCR detection of YTHDF2 binding to APC and GSK3β mRNA in LN229 cells. **J-K**: mRNA stability assay measuring APC and GSK3β mRNA half-life in YTHDF2 or PRMT6 overexpressed cells

To understand how PRMT6 facilitates glioblastoma migration, invasion, and EMT through YTHDF2, we used TCGA and CGGA databases for GSEA pathway analysis. Both PRMT6 and YTHDF2 were found to activate the Wnt-β-catenin pathway (Fig. 7A-B, Supplementary Material 5: Fig. S5A-B). Prior studies have shown that in colorectal cancer and esophageal squamous cell carcinoma, YTHDF2 activates this pathway by targeting m^6^A-modified GSK3β and APC mRNAs for degradation [52, 53]. In the absence of Wnt signaling, β-catenin is degraded by a complex that includes APC, Axin, and GSK-3β. This degradation complex phosphorylates β-catenin, leading to its ubiquitination and proteasomal degradation [54, 55]. We hypothesized that YTHDF2 in glioblastoma could bind to m^6^A sites on APC and GSK-3β mRNAs, promoting their degradation and activating the Wnt-β-catenin pathway. Western blot analysis of key Wnt-β-catenin pathway components confirmed our hypothesis. Upon YTHDF2 knockdown, β-catenin and downstream targets Cyclin D1 and c-Myc were downregulated, while p-β-catenin, APC, and GSK3β were upregulated (Fig. 7C). The opposite effects were observed when YTHDF2 was overexpressed (Supplementary Material 5: Fig. S5C). Similar results were seen with PRMT6 knockdown and overexpression (Fig. 7D, Supplementary Material 5: Fig. S5D). This indicates that PRMT6 and YTHDF2 indeed activate the Wnt-β-catenin pathway, and the regulation of this pathway by PRMT6 depends on YTHDF2 (Fig. 7E). Based on previous studies [52, 53] and predictions from the m^6^A site prediction website (http://www.cuilab.cn/sramp), we anticipated that m^6^A modification sites near the 3’ ends of APC and GSK3β mRNAs might be recognized by YTHDF2 (Fig. 7F). Further, using methylated RNA immunoprecipitation (MeRIP-qPCR), we confirmed the existence of these m^6^A sites on APC and GSK3β mRNAs (Fig. 7G, Supplementary Material 5: Fig. S5E). Overexpression of YTHDF2 significantly reduced the m^6^A modification levels at these sites on both mRNAs (Fig. 7G, Supplementary Material 5: Fig. S5E). We also investigated whether PRMT6 affects m^6^A modifications on these mRNAs and found that overexpressing PRMT6 similarly decreased m^6^A levels at these sites (Fig. 7H, Supplementary Material 5: Fig. S5F). RIP-qPCR experiments demonstrated that YTHDF2 binds to APC and GSK-3β mRNAs in glioblastoma cells (Fig. 7I, Supplementary Material 5: Fig. S5G). mRNA stability assays showed that overexpressing YTHDF2 shortened the half-life of APC and GSK-3β mRNAs, promoting their degradation (Fig. 7J, Supplementary Material 5: Fig. S5H), a phenomenon also induced by PRMT6 (Fig. 7K, Supplementary Material 5: Fig. S5I). These results confirm that YTHDF2 binds to m^6^A sites on APC and GSK-3β mRNAs, promoting their degradation and thereby activating the Wnt-β-catenin pathway. Finally, we investigated if the modulation of malignant biological behavior in glioblastoma by PRMT6 and YTHDF2 depends on the activation of the Wnt-β-catenin pathway. Adding a Wnt-β-catenin pathway activator (CHIR-99021) partially restored the migration, invasion, and EMT abilities inhibited by PRMT6 or YTHDF2 knockdown (Supplementary Material 6: Fig. S6A-D). This suggests that PRMT6 promotes glioblastoma’s malignant traits via YTHDF2-mediated activation of the Wnt-β-catenin pathway.

### The transcriptional activation of YTHDF2 by PRMT6 requires its methyltransferase activity, and inhibiting this enzymatic activity can suppress the malignant phenotype of glioma

**Fig. 8:**
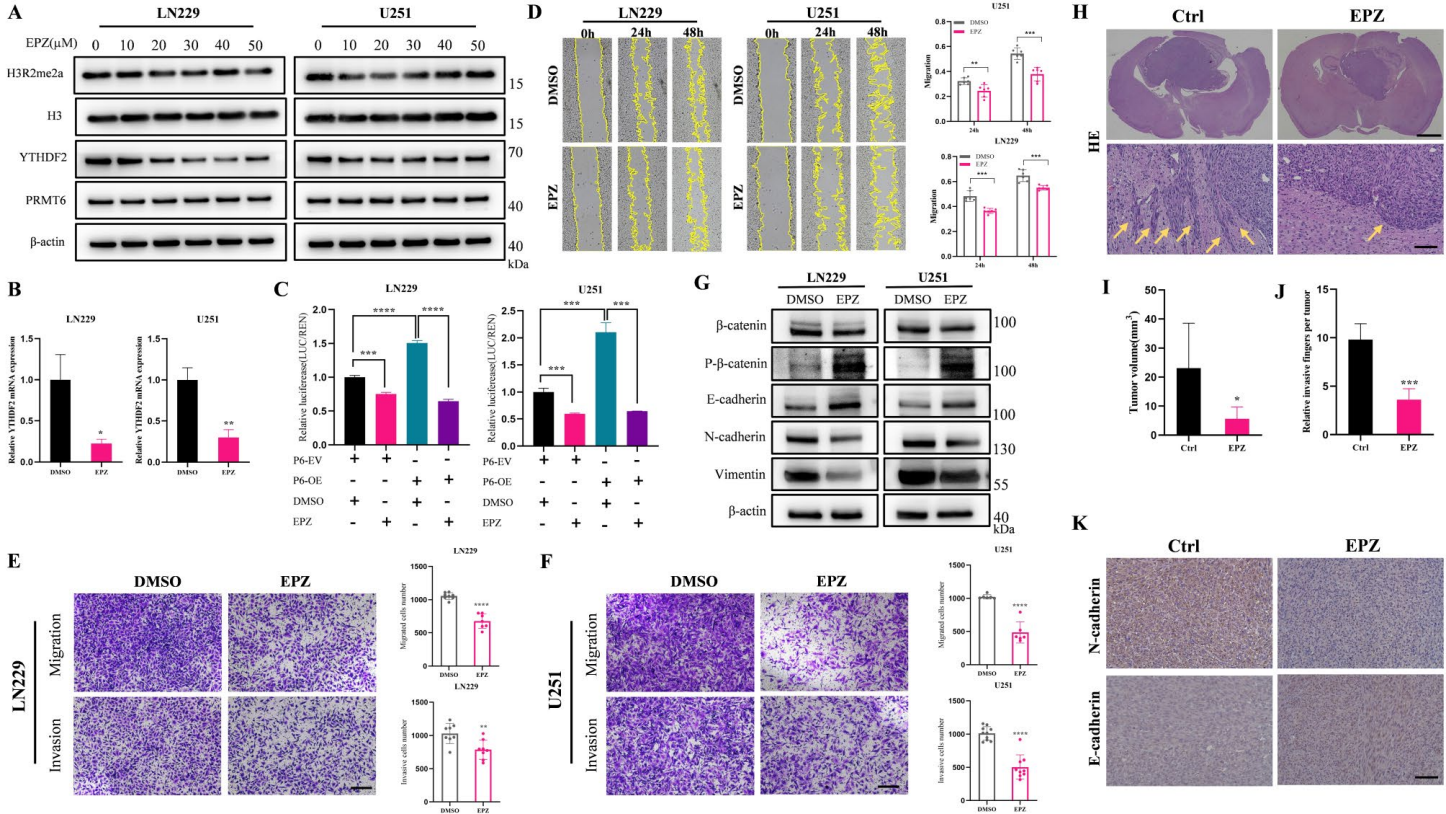
The Transcriptional Activation of YTHDF2 Mediated by PRMT6 Requires Its Methyltransferase Activity; Inhibiting This Activity Suppresses Glioma Cell Migration, Invasion, and EMT. **A**: Analysis of H3R2me2a, PRMT6, and YTHDF2 protein levels in LN229 and U251 cells treated with varying concentrations of EPZ020411 for 24 h. **B**: Assessment of YTHDF2 mRNA expression after 24-hour treatment with 20µM EPZ020411 in LN229 and U251 cells. **C**: Dual-luciferase reporter assays in LN229 or U251 cells treated with 20µM EPZ020411 for 24 h, with or without PRMT6 overexpression. **D**: Wound healing assay to evaluate cell migration in LN229 and U251 cells with or without EPZ020411 treatment. **E-F**: Transwell assays assessing cell migration and invasion with or without EPZ020411 treatment. Scale bar = 200 μm. **G**: Examination of EMT and Wnt-β-catenin pathway-related protein expression in LN229 and U251 cells treated with or without 20µM EPZ020411 for 24 h. **H**: Wild-type LN229 cells were intracranially implanted into nude mice followed by subcutaneous administration of EPZ or saline for three weeks to observe the effect of EPZ on in vivo tumorigenesis. Representative brain sections stained with H&E displayed xenograft tumors (upper panel, scale bar = 1.5 mm). In vivo invasion assays were conducted by examining the tumor margins in mouse brains (lower panel, scale bar = 100 μm). **I**: Tumor volumes for each group of mice were calculated. **J**: The relative invasive fingers of each tumor were calculated under a microscope by counting prominent and diffuse tumor tissues. **K**: Representative images of immunohistochemical staining for N-cadherin and E-cadherin in xenograft tumors from control and EPZ-treated groups of nude mice. Scale bar = 100 μm

To investigate whether PRMT6’s transcriptional activation of YTHDF2 depends on its methyltransferase activity, we utilized the specific small molecule inhibitor EPZ020411, which selectively inhibits PRMT6’s enzymatic function without affecting its expression [56]. The suitable working concentration of EPZ020411 was found to be around 20–30µM, effectively inhibiting H3R2me2a levels and subsequently suppressing YTHDF2 protein expression (Fig. 8A). Treatment with 20µM EPZ020411 for 24 h resulted in decreased mRNA expression of YTHDF2 in glioblastoma cell lines (Fig. 8B). Dual-luciferase reporter assays further confirmed that inhibiting PRMT6’s methyltransferase activity led to a reduction in luciferase activity, which was also observed in the context of PRMT6 overexpression (Fig. 8C). These results suggest the necessity of PRMT6’s methyltransferase activity for the transcriptional activation of YTHDF2. Considering that PRMT6 promotes glioblastoma progression and Wnt-β-catenin pathway activation through YTHDF2, and that inhibiting its enzymatic activity reduces YTHDF2 expression, we explored whether this inhibition could suppress malignant phenotypes in glioblastoma. The scratch assay showed significant migration inhibition in LN229 and U251 cell lines post-treatment (Fig. 8D), and Transwell assays indicated reduced migration and invasion compared to the DMSO control (Fig. 8E-F). We aimed to investigate whether EPZ020411 affects EMT and the Wnt-β-catenin pathway. Western blot results showed that inhibition of PRMT6 reduced the expression of β-catenin, vimentin, and N-cadherin, while increasing the expression of p-β-catenin and E-cadherin (Fig. 8G). Subsequently, we further validated the effect of EPZ020411 on tumors in vivo. Compared to the control group, the PRMT6 inhibitor suppressed tumor growth in vivo, and the boundary between tumor tissue and surrounding normal brain tissue in the PRMT6 inhibitor group was relatively clear (Fig. 8H-J). Immunohistochemical staining was employed to detect the expression of EMT-related proteins in mouse tumor tissues. The results showed that compared to the control group, the expression of N-cadherin was lower and the expression of E-cadherin was higher in tumors treated with the PRMT6 inhibitor (Fig. 8K). In summary, EPZ020411 can inhibit the malignant progression of glioblastoma both in vitro and in vivo.

### PRMT6 promotes glioma invasive growth and EMT via YTHDF2 in vivo

**Fig. 9:**
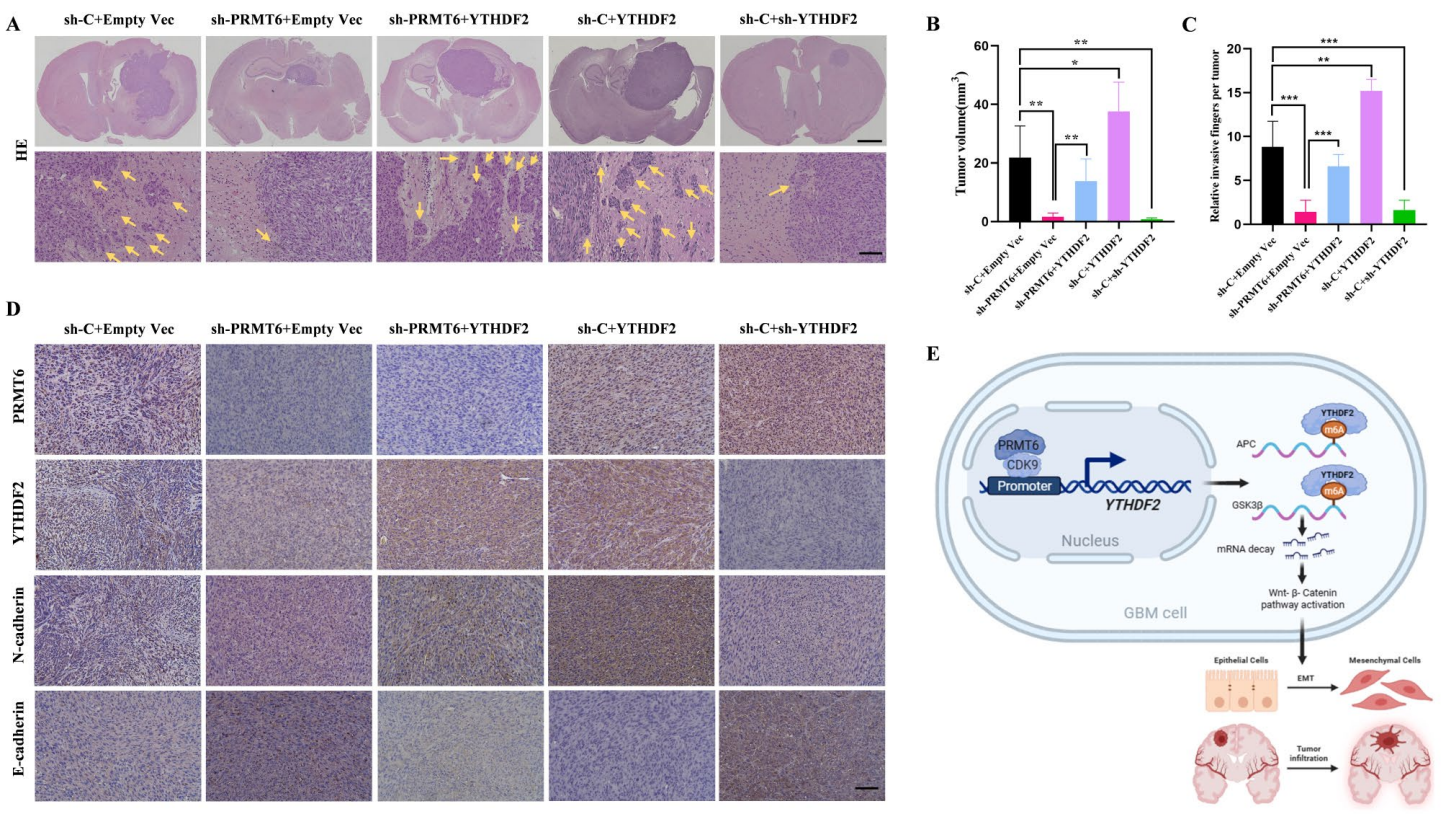
PRMT6 Promotes Glioma Invasive Growth and EMT in an Orthotopic Xenograft Model via YTHDF2. **A**: A nude mouse intracranial tumorigenesis assay was conducted using sh-C + Empty Vec, sh-PRMT6 + Empty Vec, sh-PRMT6 + YTHDF2, sh-C + YTHDF2, and sh-C + sh-YTHDF2 cells. Representative H&E-stained brain sections show orthotopic xenografts (top images, scale bar = 1.5 mm). Tumor margins in mouse brains were observed for in vivo invasion assessment (bottom images, scale bar = 100 μm). **B**: Tumor volumes were calculated for each mouse group. **C**: The relative invasive fingers of each tumor were calculated microscopically by counting protruding and diffused tumor tissues. **D**: Representative immunohistochemical staining images of PRMT6, YTHDF2, N-cadherin, and E-cadherin in mouse tumor tissues. Scale bar = 100 μm

Finally, we further validated the role of the PRMT6-YTHDF2 axis in promoting glioblastoma malignancy in vivo. Xenografts of LN229 cells confirmed that tumors from the sh-C + Empty Vec group exhibited significant infiltration into normal brain tissue with unclear boundaries, while the sh-PRMT6 + Empty Vec group markedly suppressed tumor growth, and the boundary between tumor tissue and surrounding normal brain tissue was relatively clear (Fig. 9A-C). However, re-expression of YTHDF2 on the basis of PRMT6 knockdown (sh-PRMT6 + YTHDF2 group) partially counteracted the above effects of PRMT6 knockdown (Fig. 9A-C). Similarly, compared to the control group, tumors in the sh-C + YTHDF2 group had larger volumes with unclear boundaries between tumor tissue and surrounding normal brain tissue, while the opposite was observed in the sh-C + sh-YTHDF2 group (Fig. 9A-C). Immunohistochemical staining was used to detect the expression of relevant proteins in mouse tumor tissues. The results showed that compared to the control group, the expression of PRMT6, YTHDF2, and N-cadherin was lower, while E-cadherin expression was higher in tumors from the sh-PRMT6 + Empty Vec group (Fig. 9D). However, in tumors from the sh-PRMT6 + YTHDF2 group, N-cadherin expression was re-upregulated, and E-cadherin expression was downregulated (Fig. 9D). Similarly, compared to the control group, N-cadherin expression was higher, while E-cadherin expression was lower in tumors from the sh-C + YTHDF2 group, and the opposite was observed in the sh-C + sh-YTHDF2 group (Fig. 9D). These experimental results confirm that PRMT6 and YTHDF2 promote the EMT and invasive phenotype of glioblastoma in vivo, and PRMT6 promotes tumor malignancy in vivo by regulating the expression of YTHDF2.

## Discussion

GBM often exhibits infiltrative growth, blurring the boundaries with surrounding brain tissues, making curative resection challenging. Consequently, nearly all GBM patients experience postoperative recurrence [57]. Studies suggest that tumor cells, after undergoing EMT, acquire the potential for migration and invasion [58], making EMT a key factor in the infiltrative growth of gliomas. Based on integrated transcriptomic and genomic data analysis, GBM is classified into mesenchymal (MES), classical (CL), and proneural (PN) subtypes [49]. The MES subtype is closely associated with the invasive phenotype of gliomas, and non-MES subtype GBMs often acquire MES characteristics upon recurrence, a process similar to tumor cells undergoing EMT to gain enhanced invasive capabilities [49, 59]. Previous studies have indicated that EMT is a reversible and dynamic process likely induced by epigenetic changes driven by the tumor microenvironment, rather than genetic changes [60–62]. In this study, we reveal that the PRMT6-YTHDF2-Wnt-β-Catenin axis promotes migration, invasion, and EMT in gliomas, both in vivo and in vitro (Fig. 9E).

PRMT6, as an epigenetic mediator, mainly catalyzes the asymmetric dimethylation of arginine residues on histones and non-histone proteins. Many studies have reported on the role of PRMT6 in various cancers, where its expression is significantly increased in most tumors, indicating its crucial role in tumorigenesis. In glioma, Huang et al.‘s study showed that PRMT6 methylates RCC1, thereby regulating mitosis, tumorigenicity, and radiotherapy response in glioblastoma stem cells [32]. Wang et al. demonstrated that PRMT6, via H3R2me2a, promotes CDC20 transcription and mediates CDKN1B degradation, thereby facilitating glioma proliferation and cell cycle regulation [35]. These suggest PRMT6’s significant role in glioma development and progression. However, the specific mechanisms by which PRMT6 regulates glioma migration, invasion, and EMT are not yet reported. Recent literature indicates that PRMT6 promotes breast cancer migration and distant metastasis by methylating STAT3, thereby regulating the IL-6/STAT3 pathway [63]. In our study, we confirm that PRMT6 promotes glioma cell migration, invasion, and EMT by transcriptionally activating YTHDF2. PRMT6 cannot bind directly to DNA; as a transcriptional regulator, it mainly exerts transcriptional repression through H3R2me2a modification on target genes [17, 21, 22, 24]. However, literature reports that PRMT6 can act as a co-factor for transcription factors, being recruited to target genes to form part of a multi-component transcription complex, thereby activating expression of these target genes [24–26]. We confirmed that the transcriptional regulator CDK9 interacts with PRMT6 and recruits it to the promoter region of YTHDF2. To confirm the co-regulatory role of PRMT6 and CDK9 on YTHDF2 transcriptional activation, we overexpressed PRMT6 and CDK9 simultaneously in HEK-293T cells for dual-luciferase reporter assays. Surprisingly, co-expression did not result in additive effects on luciferase activity (Fig. 4F), possibly due to saturation of regulatory complexes, competition for limited binding sites, or the presence of other regulatory factors that might counteract the function of transcription factors and co-factors. Thus, the impact of simultaneous overexpression of transcription factors and co-factors on target gene expression can vary due to environmental factors and complex regulatory networks. However, we found that luciferase activity decreased when CDK9 was overexpressed on a background of PRMT6 knockdown (Fig. 4I), and similarly when PRMT6 was overexpressed on a background of CDK9 knockdown (Fig. 4J). This suggests a mutual dependence and collaborative regulation of YTHDF2 transcriptional activation by PRMT6 and CDK9. CDK9 is widely expressed in human tissues, forming the positive transcription elongation factor b (P-TEFb) complex with Cyclin T1. As a core component of P-TEFb, CDK9 plays a crucial role in regulating transcriptional elongation [64, 65]. P-TEFb phosphorylates RNA polymerase II (RNA Pol II), releasing paused RNA Pol II from promoter-proximal sites to continue transcription elongation and produce mature mRNA [65–68]. Qiu et al.‘s study showed that the YY1-CDK9 complex and transcriptional elongation complex co-regulate m^6^A programmatic expression. Knockdown of CDK9 or selective CDK9 inhibitors reduced YTHDF2 expression levels in glioblastoma stem cells [69], consistent with our findings (Fig. 4G, Supplementary Material 3: Fig. S3E). To further confirm whether PRMT6’s transcriptional activation of YTHDF2 depends on its methyltransferase function, we used the specific inhibitor EPZ020411, which inhibits PRMT6’s methyltransferase function without affecting its expression. Our findings suggest that PRMT6’s transcriptional activation of YTHDF2 depends on its methyltransferase activity. It should be noted that we are currently unclear whether PRMT6 directly methylates CDK9 or methylates transcriptional regulators related to CDK9, thus affecting CDK9’s function in promoting transcriptional elongation. The specific molecular mechanisms require further investigation by other members of our team. Encouragingly, our in vitro and in vivo study reveals that EPZ020411 effectively inhibits glioma cell migration, invasion, and EMT. Huang et al.‘s study indicates that EPZ020411 can cross the blood-brain barrier [32], suggesting promising anti-cancer potential for EPZ020411 against GBM.

YTHDF2, as a key m^6^A reader protein, primarily functions by binding to m^6^A -modified target mRNAs and promoting their degradation. Previous literature has confirmed the upregulation of YTHDF2 in glioma and its promotion of glioma development and progression through various molecular mechanisms [7, 46, 47]. Our study validates that PRMT6 transcriptionally activates YTHDF2, thereby promoting malignant phenotypes in glioma, a process that depends on PRMT6’s methyltransferase activity (Fig. 8). This suggests that the state of protein arginine methylation affects YTHDF2 expression. To further clarify how PRMT6 transcriptionally activates YTHDF2 and then promotes malignant phenotypes in glioma, we conducted GSEA pathway analysis. The results suggest that both PRMT6 and YTHDF2 can activate the Wnt-β-Catenin pathway, implicating its involvement in the regulation of glioma migration, invasion, and EMT by PRMT6 and YTHDF2. Several studies have found that activated Wnt-β-Catenin signaling is closely related to tumor cell migration, invasion, and EMT. β-Catenin, sequestered in the cytoplasm by E-cadherin, is released and translocated into the nucleus when E-cadherin is downregulated [50, 70]. Once in the nucleus, β-catenin binds with transcription factors TCF/LEF, inducing the expression of EMT-related activators like Twist1, Slug, and Snail1 [71, 72]. These transcription factors suppress the expression of epithelial genes and promote mesenchymal gene expression, driving the cell towards a mesenchymal state and activating EMT. In previous studies, Wang et al. [53] and Li et al. [52] demonstrated in esophageal squamous cell carcinoma and colorectal cancer, respectively, that YTHDF2 binds to m^6^A -modified APC and GSK3β mRNA, promoting their degradation and activating the Wnt-β-Catenin pathway. In our study, we are the first to demonstrate in glioma cells that YTHDF2 binds to m^6^A -modified APC and GSK3β mRNA, promoting their degradation and thereby activating the Wnt-β-Catenin pathway. Moreover, we show that the impact of PRMT6 and YTHDF2 on glioma migration, invasion, and EMT depends on the activation of the Wnt-β-Catenin pathway (Supplementary Material 6: Fig. S6). While our study did not explore the overall impact of PRMT6 on m^6^A modifications in glioma cells, we found that PRMT6 affects the m^6^A modification of APC and GSK3β mRNA, as overexpression of PRMT6 reduces the m^6^A levels on these mRNAs (Fig. 7H, Supplementary Material 5: Fig. S5F). This links protein methylation modifications with RNA methylation mechanisms, providing new insights into the epigenetic regulation of glioma development and progression. In summary, our study confirms that PRMT6, as a co-factor of transcription factors, collaborates with CDK9 to promote YTHDF2 expression, thereby suppressing the expression of YTHDF2 target genes APC and GSK3β, and activating the Wnt-β-Catenin pathway. These findings reveal the role of the PRMT6-YTHDF2-Wnt-β-Catenin axis in the malignant phenotype of GBM, offering potential effective therapeutic targets for GBM treatment.

## Conclusions

This investigation elucidates the activation of the Wnt-β-Catenin pathway by PRMT6 through transcriptional upregulation of YTDHF2, highlighting the significance of the PRMT6-YTHDF2-Wnt-β-Catenin axis in facilitating GBM’s migration, invasion, and EMT in both in vitro and in vivo contexts. Additionally, it demonstrates the efficacy of PRMT6 small molecule inhibitors in suppressing these malignant characteristics in vitro. This study bridges the understanding of protein and RNA methylation mechanisms, offering novel perspectives for exploring epigenetic regulation in GBM pathogenesis and progression.

### Electronic supplementary material

Below is the link to the electronic supplementary material.

Supplementary Material 1: Figure S1

Supplementary Material 2: Figure S2

Supplementary Material 3: Figure S3

Supplementary Material 4: Figure S4

Supplementary Material 5: Figure S5

Supplementary Material 6: Figure S6

Supplementary Material 7: Table S1

Supplementary Material 8: Table S2

## Data Availability

The datasets generated and/or analyzed during the current study are available in UCSC Xena (http://xena.ucsc.edu/) and CGGA (http://www.cgga.org.cn).

## Abbreviations

IDH1: isocitrate dehydrogenase 1
MGMT: O6-methylguanine-DNA methyltransferase
EGFR: epidermal growth factor receptor
PRMT6: Protein arginine methyltransferase 6
YTHDF2: YTH domain family member 2
GBM: glioblastoma multiforme
NBT: normal brain tissue
TCGA: The Cancer Genome Atlas
GTEx: The Genotype-Tissue Expression
CGGA: Chinese Glioma Genome Atlas
GSEA: Gene Set Enrichment Analysis
MES: Mesenchymal
CL: Classical
PN: Proneural
qPCR: Quantitative Real-Time PCR
IHC: Immunohistochemistry
EMT: Epithelial-Mesenchymal Transition
P-TEFb: positive transcription elongation factor b
