# Supplementary figures and images for: PRMT6-mediated transcriptional activation of ythdf2 promotes glioblastoma migration, invasion, and emt via the wnt–β-catenin pathway
Source: J Exp Clin Cancer Res. 2024 Apr 18;43:116. doi: 10.1186/s13046-024-03038-3 (PMC11025288; doi:10.1186/s13046-024-03038-3)

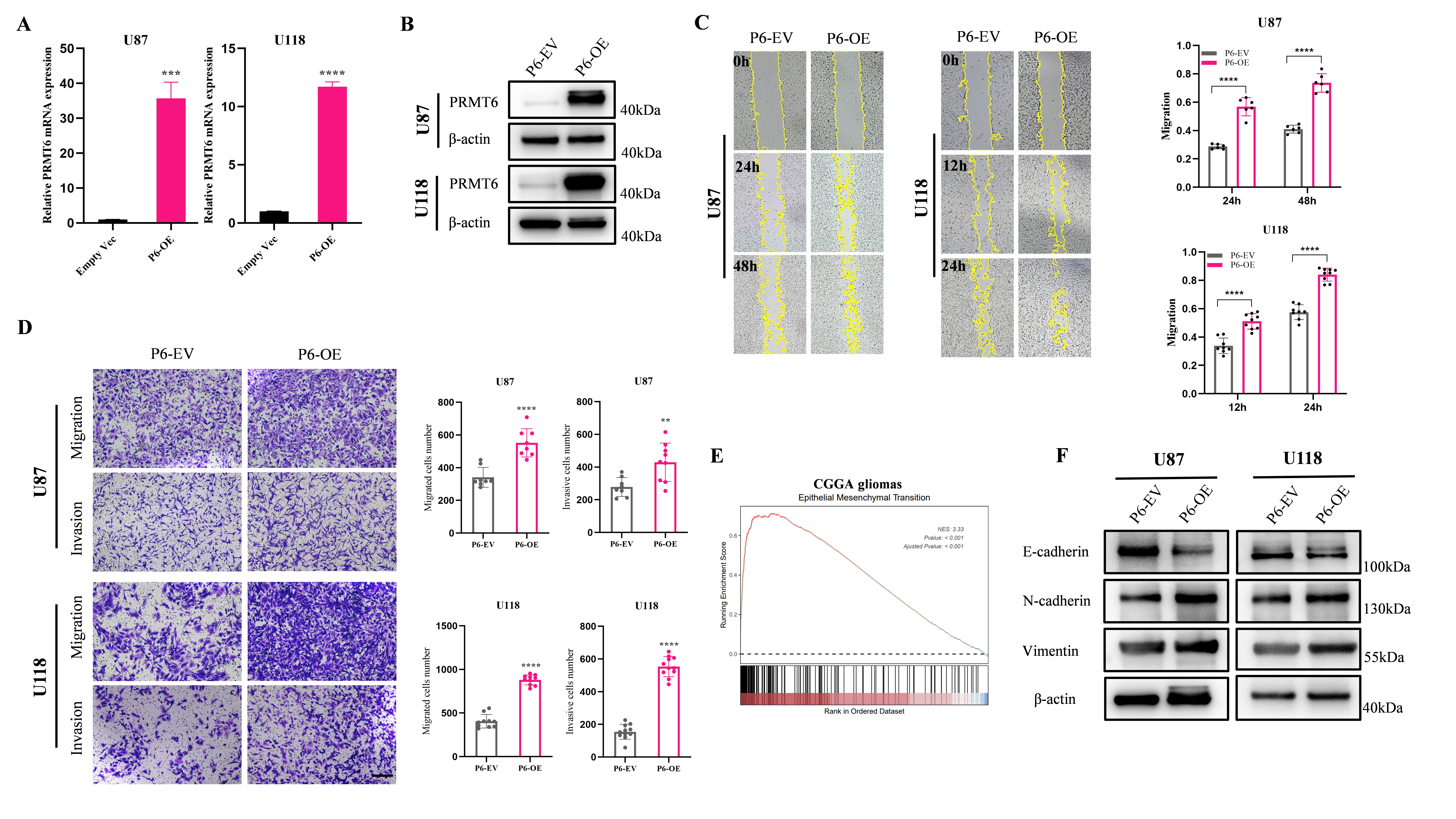

Supplement: Supplementary file 1 — Supplementary Material 1: Figure S1 [file 13046_2024_3038_MOESM1_ESM.jpg]

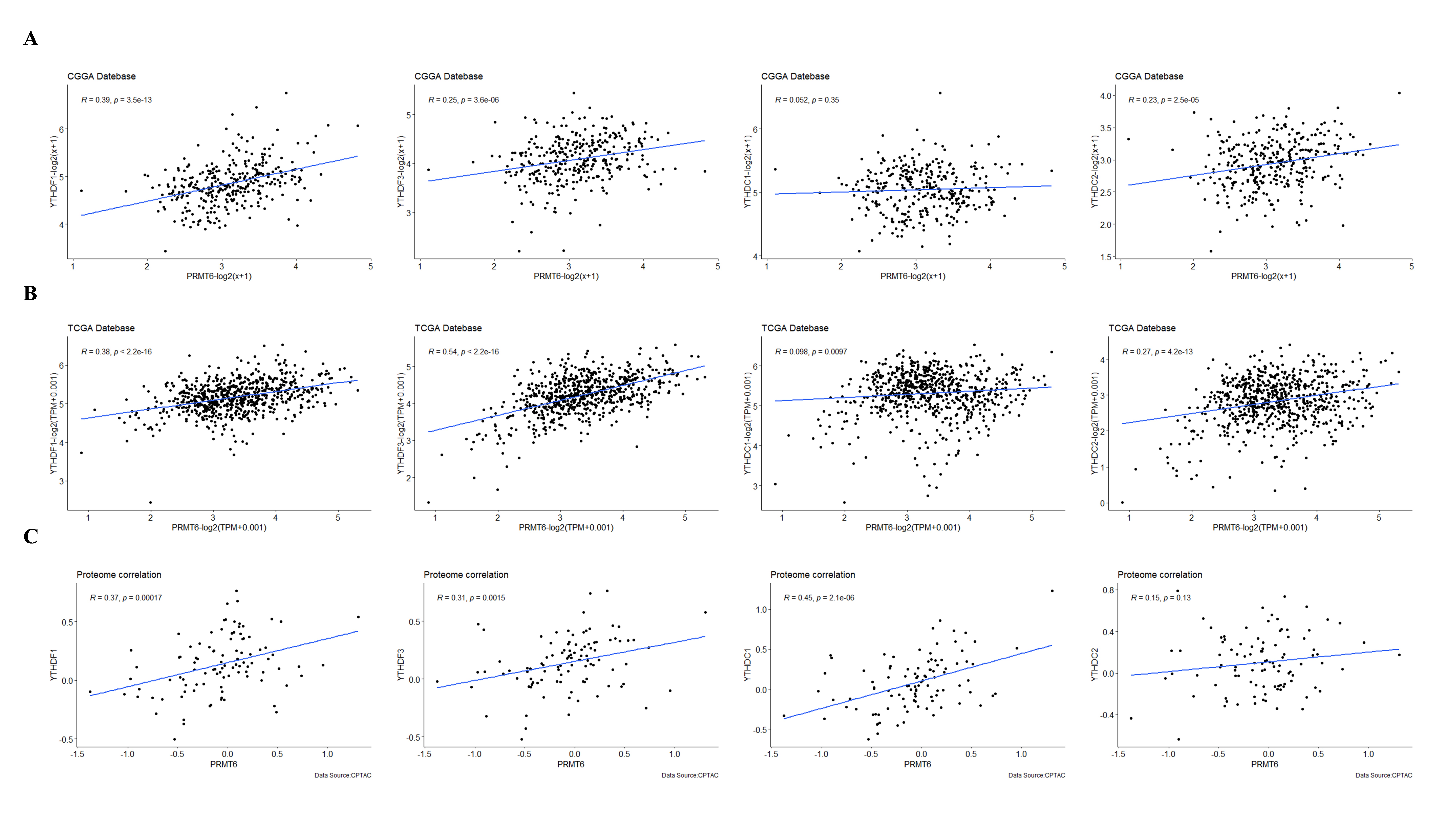

Supplement: Supplementary file 2 — Supplementary Material 2: Figure S2 [file 13046_2024_3038_MOESM2_ESM.jpg]

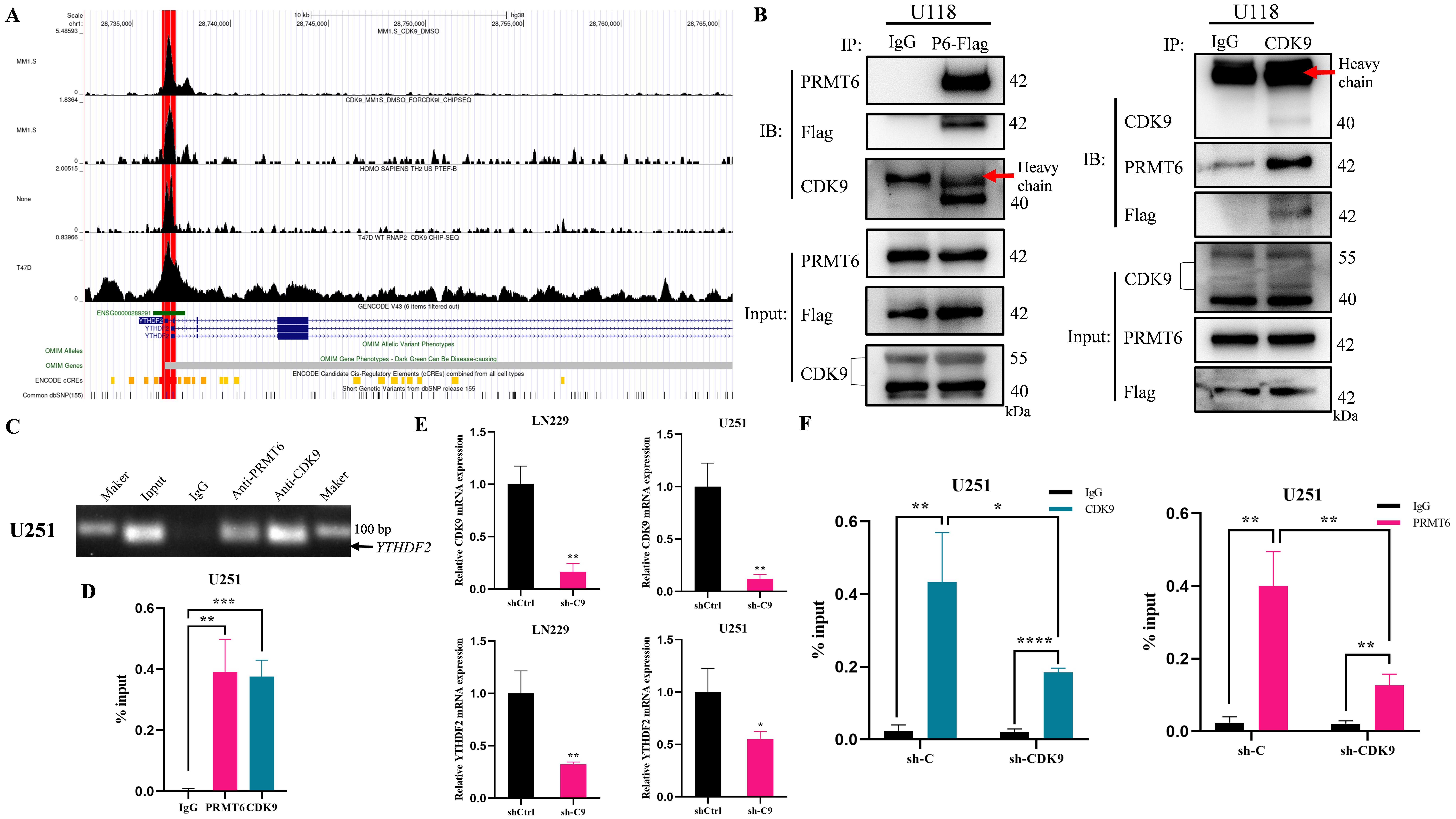

Supplement: Supplementary file 3 — Supplementary Material 3: Figure S3 [file 13046_2024_3038_MOESM3_ESM.jpg]

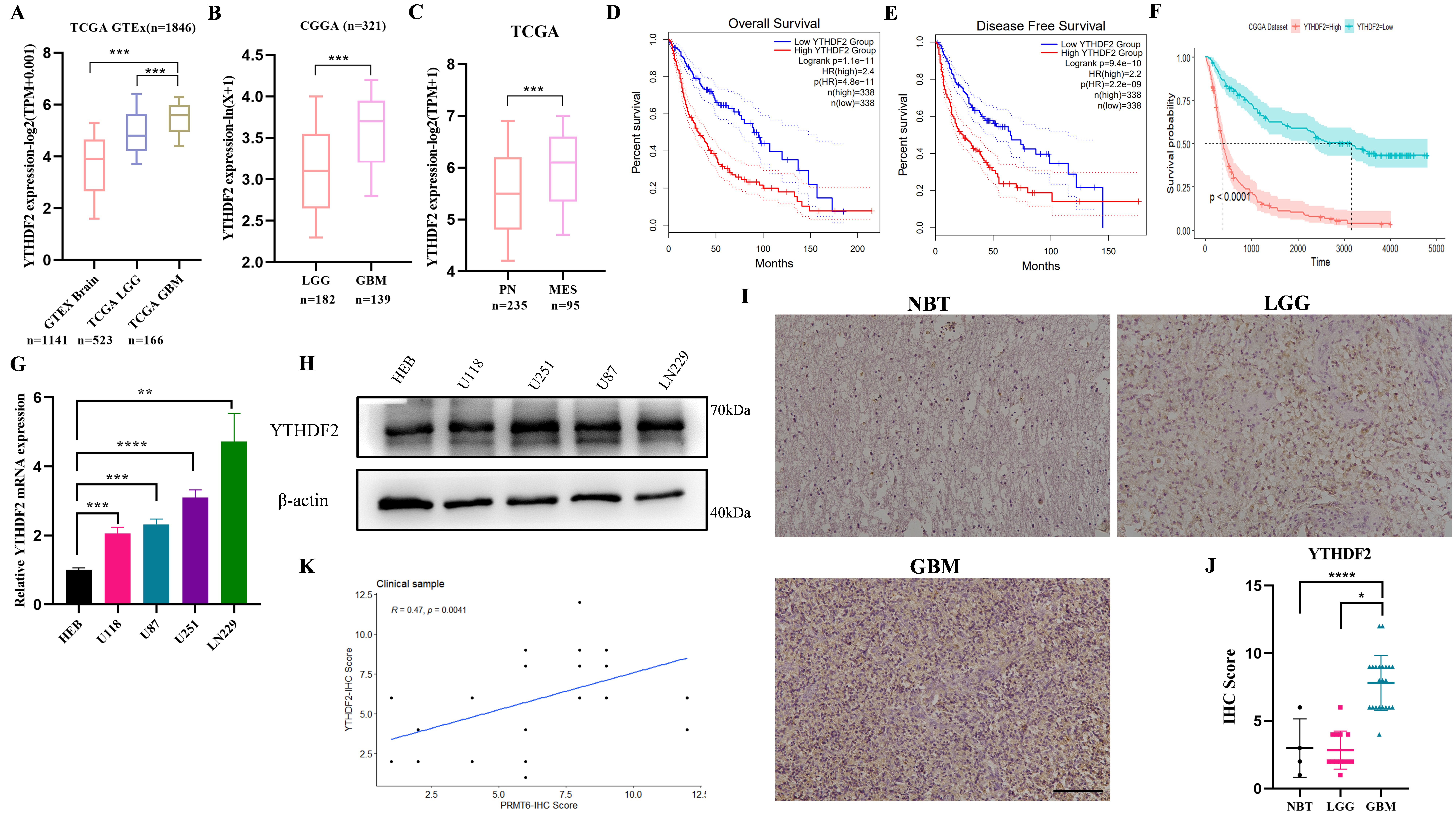

Supplement: Supplementary file 4 — Supplementary Material 4: Figure S4 [file 13046_2024_3038_MOESM4_ESM.jpg]

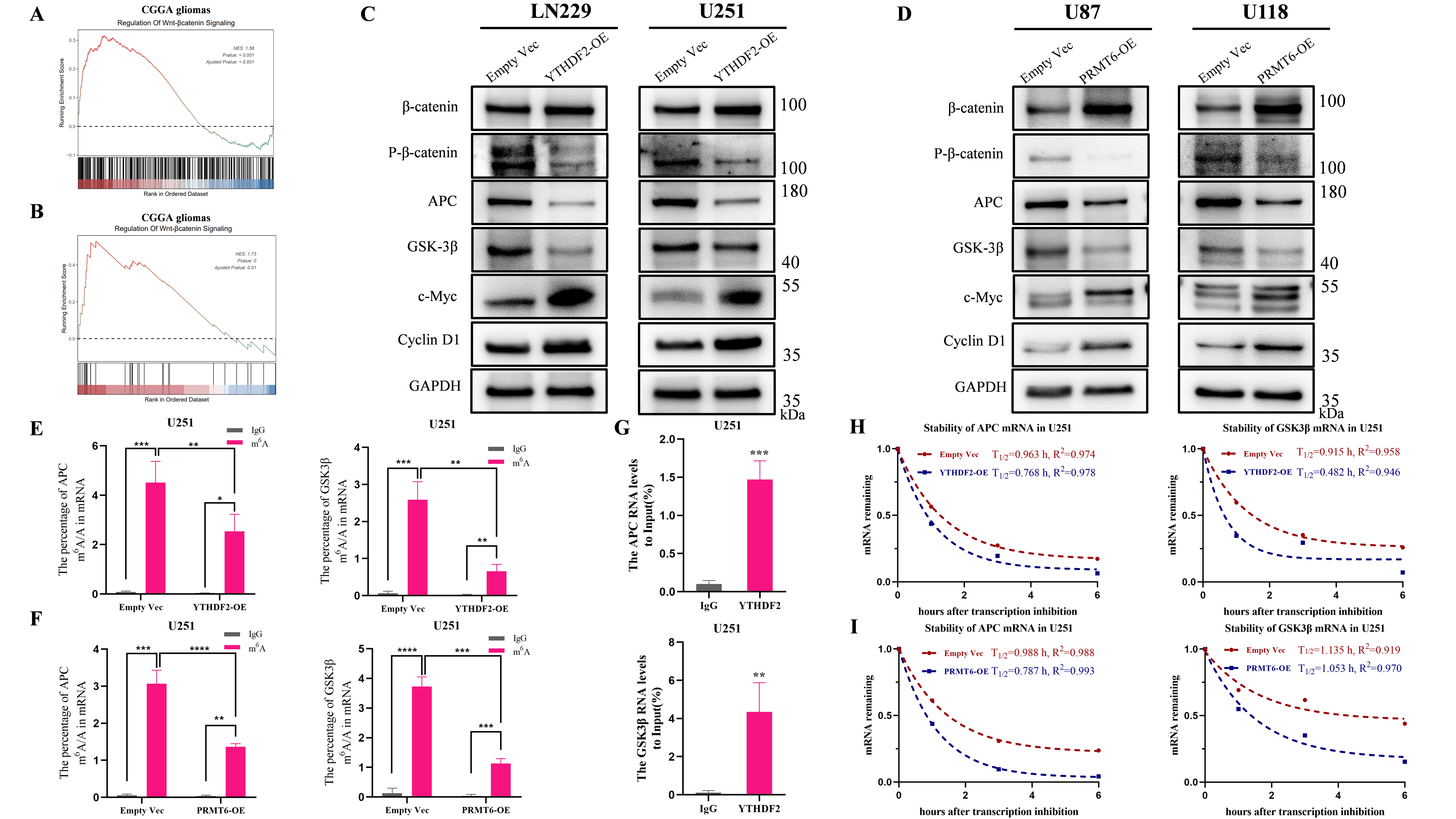

Supplement: Supplementary file 5 — Supplementary Material 5: Figure S5 [file 13046_2024_3038_MOESM5_ESM.jpg]

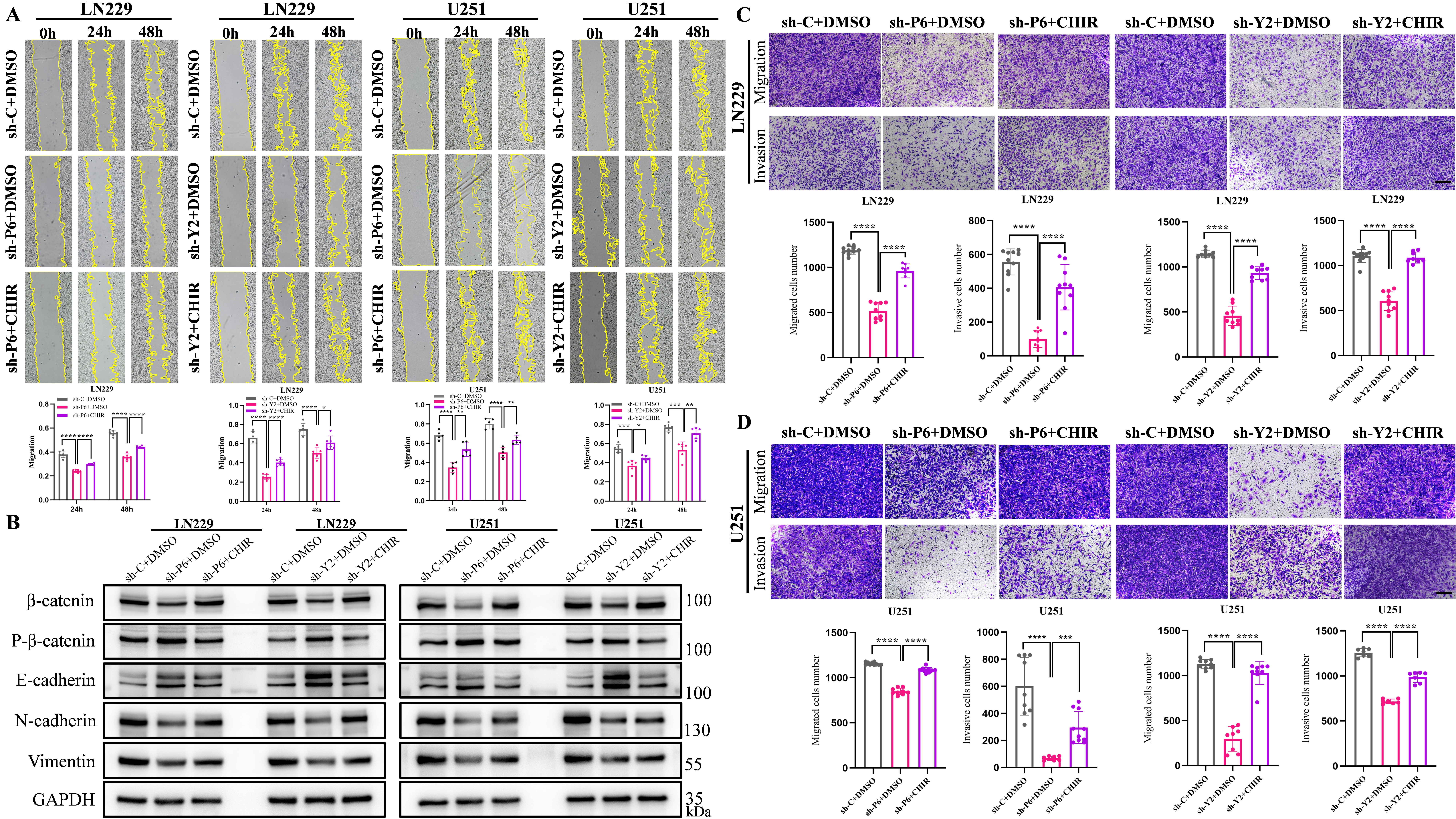

Supplement: Supplementary file 6 — Supplementary Material 6: Figure S6 [file 13046_2024_3038_MOESM6_ESM.jpg]
